# Data-driven modelling of tau pathology reveals distinct progressive supranuclear palsy subtypes

**DOI:** 10.1093/brain/awag131

**Published:** 2026-04-13

**Authors:** Patrick W Cullinane, Jacy Bezerra Parmera, Hemanth Nelvagal, Toby Curless, Leckhna Paras Chajed, Sarah Wrigley, Walter Sifontes Valladares, Maggie Burrows, Kirsten Ebanks, Lesley Wu, Lawrence P Binding, Jaime Anton, Tamas Revesz, Raquel Real, David P Vaughan, Edwin Jabbari, Huw R Morris, Sebastian Brandner, Alexandra L Young, Glen Hoti, Yangxinrui Ma, Mariam Bechtawi, Nancy Chiraki, Eduardo de Pablo-Fernández, Yau Mun Lim, Thomas T Warner, Zane Jaunmuktane

**Affiliations:** Department of Clinical and Movement Neurosciences, UCL Queen Square Institute of Neurology, University College London, London WC1N 3BG, UK; Queen Square Brain Bank for Neurological Disorders, UCL Queen Square Institute of Neurology, London WC1N 1PJ, UK; Reta Lila Weston Institute of Neurological Studies, UCL Queen Square Institute of Neurology, London WC1N 1PJ, UK; Queen Square Movement Disorders Centre, UCL Queen Square Institute of Neurology, London WC1N 3BG, UK; Queen Square Brain Bank for Neurological Disorders, UCL Queen Square Institute of Neurology, London WC1N 1PJ, UK; Department of Neurology, Hospital das Clínicas, Faculdade de Medicina da Universidade de São Paulo (HC-FMUSP), São Paulo 05402 000, Brazil; Department of Clinical and Movement Neurosciences, UCL Queen Square Institute of Neurology, University College London, London WC1N 3BG, UK; Queen Square Brain Bank for Neurological Disorders, UCL Queen Square Institute of Neurology, London WC1N 1PJ, UK; Queen Square Brain Bank for Neurological Disorders, UCL Queen Square Institute of Neurology, London WC1N 1PJ, UK; Queen Square Brain Bank for Neurological Disorders, UCL Queen Square Institute of Neurology, London WC1N 1PJ, UK; Department of Clinical and Movement Neurosciences, UCL Queen Square Institute of Neurology, University College London, London WC1N 3BG, UK; Reta Lila Weston Institute of Neurological Studies, UCL Queen Square Institute of Neurology, London WC1N 1PJ, UK; Queen Square Brain Bank for Neurological Disorders, UCL Queen Square Institute of Neurology, London WC1N 1PJ, UK; Department of Clinical and Movement Neurosciences, UCL Queen Square Institute of Neurology, University College London, London WC1N 3BG, UK; Queen Square Brain Bank for Neurological Disorders, UCL Queen Square Institute of Neurology, London WC1N 1PJ, UK; Department of Clinical and Movement Neurosciences, UCL Queen Square Institute of Neurology, University College London, London WC1N 3BG, UK; Queen Square Brain Bank for Neurological Disorders, UCL Queen Square Institute of Neurology, London WC1N 1PJ, UK; Department of Clinical and Movement Neurosciences, UCL Queen Square Institute of Neurology, University College London, London WC1N 3BG, UK; UCL Hawkes Institute, Department of Computer Science, University College London, London WC1V 6LJ, UK; Department of Clinical and Movement Neurosciences, UCL Queen Square Institute of Neurology, University College London, London WC1N 3BG, UK; Queen Square Brain Bank for Neurological Disorders, UCL Queen Square Institute of Neurology, London WC1N 1PJ, UK; Queen Square Brain Bank for Neurological Disorders, UCL Queen Square Institute of Neurology, London WC1N 1PJ, UK; Department of Neurodegenerative Disease, UCL Queen Square Institute of Neurology, London WC1N 3BG, UK; Department of Clinical and Movement Neurosciences, UCL Queen Square Institute of Neurology, University College London, London WC1N 3BG, UK; Queen Square Movement Disorders Centre, UCL Queen Square Institute of Neurology, London WC1N 3BG, UK; Department of Clinical and Movement Neurosciences, UCL Queen Square Institute of Neurology, University College London, London WC1N 3BG, UK; Department of Clinical and Movement Neurosciences, UCL Queen Square Institute of Neurology, University College London, London WC1N 3BG, UK; Department of Clinical and Movement Neurosciences, UCL Queen Square Institute of Neurology, University College London, London WC1N 3BG, UK; Queen Square Movement Disorders Centre, UCL Queen Square Institute of Neurology, London WC1N 3BG, UK; Department of Neurodegenerative Disease, UCL Queen Square Institute of Neurology, London WC1N 3BG, UK; Division of Neuropathology, National Hospital for Neurology and Neurosurgery, University College London NHS Foundation Trust, London WC1N 3BG, UK; UCL Hawkes Institute, Department of Computer Science, University College London, London WC1V 6LJ, UK; Queen Square Brain Bank for Neurological Disorders, UCL Queen Square Institute of Neurology, London WC1N 1PJ, UK; Queen Square Brain Bank for Neurological Disorders, UCL Queen Square Institute of Neurology, London WC1N 1PJ, UK; Department of Clinical and Movement Neurosciences, UCL Queen Square Institute of Neurology, University College London, London WC1N 3BG, UK; Queen Square Brain Bank for Neurological Disorders, UCL Queen Square Institute of Neurology, London WC1N 1PJ, UK; Department of Clinical and Movement Neurosciences, UCL Queen Square Institute of Neurology, University College London, London WC1N 3BG, UK; Queen Square Brain Bank for Neurological Disorders, UCL Queen Square Institute of Neurology, London WC1N 1PJ, UK; Department of Clinical and Movement Neurosciences, UCL Queen Square Institute of Neurology, University College London, London WC1N 3BG, UK; Queen Square Brain Bank for Neurological Disorders, UCL Queen Square Institute of Neurology, London WC1N 1PJ, UK; Reta Lila Weston Institute of Neurological Studies, UCL Queen Square Institute of Neurology, London WC1N 1PJ, UK; Centre for Preventive Neurology, Wolfson Institute of Population Health, Queen Mary University London, London EC1M 6BQ, UK; Department of Neurodegenerative Disease, UCL Queen Square Institute of Neurology, London WC1N 3BG, UK; Division of Neuropathology, National Hospital for Neurology and Neurosurgery, University College London NHS Foundation Trust, London WC1N 3BG, UK; Department of Clinical and Movement Neurosciences, UCL Queen Square Institute of Neurology, University College London, London WC1N 3BG, UK; Queen Square Brain Bank for Neurological Disorders, UCL Queen Square Institute of Neurology, London WC1N 1PJ, UK; Reta Lila Weston Institute of Neurological Studies, UCL Queen Square Institute of Neurology, London WC1N 1PJ, UK; Queen Square Movement Disorders Centre, UCL Queen Square Institute of Neurology, London WC1N 3BG, UK; Department of Clinical and Movement Neurosciences, UCL Queen Square Institute of Neurology, University College London, London WC1N 3BG, UK; Queen Square Brain Bank for Neurological Disorders, UCL Queen Square Institute of Neurology, London WC1N 1PJ, UK; Reta Lila Weston Institute of Neurological Studies, UCL Queen Square Institute of Neurology, London WC1N 1PJ, UK; Division of Neuropathology, National Hospital for Neurology and Neurosurgery, University College London NHS Foundation Trust, London WC1N 3BG, UK

**Keywords:** tauopathy, phenotypic heterogeneity, machine learning, disease progression modelling

## Abstract

Progressive supranuclear palsy (PSP) is a heterogeneous neurodegenerative disease characterized by the accumulation of misfolded 4-repeat tau within neurons and glial cells. There are limited longitudinal data on pathologically confirmed PSP patients with phenotypes other than classic Richardson’s syndrome (RS) and the pathomechanisms responsible for the broad variability in clinical phenotype and progression are not well understood. An unresolved question in this context is whether distinct spatiotemporal patterns of tau pathology propagation exist within the clinicopathological spectrum of PSP.

We included 241 consecutive, pathologically confirmed patients with PSP from the Queen Square Brain Bank for Neurological Disorders (2010–2022). Phenotyping was performed based on clinical features present within the first 3 years from symptom onset according to the Movement Disorder Society (MDS) criteria, and specific clinical features and disease milestones were recorded. Genotyping was performed using Illumina NeuroBooster and NeuroChip arrays and *MAPT* haplotype, *APOE* genotype, *TRIM11* rs564309 and *SLC2A13* rs2242367 single nucleotide polymorphism data were collated. Tissue sections from eight brain regions, mounted on glass slides, were immunostained for hyperphosphorylated tau and digitized using whole-slide scanning. Forty-one anatomical regions of interest were manually segmented, and total tau pathology burden was quantified using an automated, machine learning-based algorithm. The associations between survival and both clinicogenetic features and regional tau pathology burden were modelled using Cox regression and generalized linear models, respectively, and the Subtype and Stage Inference (SuStaIn) algorithm was used to identify subgroups with distinct progression patterns.

We have identified: (i) several clinical predictors of survival in PSP and the relationship between regional tau pathology burden and survival; (ii) novel anatomical reference standards for the expected distribution of tau pathology across MDS-defined PSP phenotypes, including region-specific white matter involvement in patients with corticobasal syndrome and speech/language variants; (iii) associations potentially linking biological sex, *MAPT* haplotype and TAR DNA-binding protein 43 (TDP-43) co-pathology to clinical phenotype and regional tau pathology burden; (iv) patterns of covariance in regional tau pathology implicating inter-regional connectivity in tau spreading; and (v) three distinct spatiotemporal patterns of tau pathology progression: one characterized by initial involvement of subcortical grey matter followed by rostral spread to cortical regions and two characterized by early, simultaneous involvement of subcortical grey matter and cortical regions. Taken together, these results indicate that PSP clinicopathological heterogeneity is mediated by propagation of tau pathology along anatomically connected networks and via intrinsic regional susceptibility mechanisms, possibly influenced by sex, genetic factors and co-pathology.

## Teaser

Cullinane *et al.* used machine learning to quantify tau pathology in brain tissue from more than 200 individuals with PSP. Data-driven modelling identified three PSP subtypes with different progression patterns and showed that sex, genetic variation, and co-pathology influence tau distribution and clinical phenotype.

**See Thal and Schaeverbeke. (https://doi.org/10.1093/brain/awag271) for a scientific commentary on this article.**

## Introduction

Progressive supranuclear palsy (PSP) is characterized by the accumulation of misfolded 4-repeat tau within neurons and glial cells forming hallmark astrocytic inclusions in the latter, referred to as tufted astrocytes.^1,2^ It is a highly heterogeneous neurodegenerative disease with considerable variability in clinical phenotype and progression, leading to challenges for accurate early diagnosis, biomarker discovery and therapeutic trials.^3^ There are limited longitudinal data on pathologically confirmed PSP patients, particularly those with non-Richardson’s syndrome (RS) clinical phenotypes.^3,4^ While clinical phenotype broadly corresponds to the anatomical distribution of tau pathology,^5-10^ large-scale quantitative assessment of regional tau pathology across PSP clinical phenotypes is lacking, and the relationship between tau pathology burden in specific brain areas and clinical progression is not well understood. The relative contributions and interactions between cell-autonomous (intrinsic susceptibility to protein misfolding) and non-autonomous (‘prion-like’ trans-synaptic spreading) mechanisms of tau pathology propagation in PSP also require further clarification. The current system for the pathological staging of PSP-RS emphasizes a spatiotemporal pattern of tau propagation whereby tau accumulation begins in the pallido-nigro-luysian axis followed by subsequent spread rostrally to the neocortex and caudally to the cerebellum,^5^ while *in vivo* work using ^18^F-Florzolotau PET imaging and data-driven modelling indicates that there is an additional pattern of tau pathology progression in some patients characterized by early simultaneous involvement of cortical and pallido-nigro-luysian regions.^11^ Limitations of previous clinicopathological studies include non-standardized clinical phenotyping,^12,13^ the use of semi-quantitative pathology assessments that are insensitive to subtle differences in tau pathology and do not account for tau burden in the subcortical white matter,^5^ as well as comparatively small sample sizes.^14^ In this study, we aimed to evaluate clinical progression in a large cohort of clinically, genetically and pathologically well-characterized PSP patients, half of whom had clinical phenotypes other than PSP-RS. Using a machine learning-based digital pathology algorithm to generate quantitative measurements of regional tau pathology, we also aimed to determine patterns of tau pathology propagation through data-driven disease progression modelling.^15^ The overall objective of this work is to uncover clinical, genetic and pathological factors that drive phenotypic diversity and disease progression in PSP in order to improve patient stratification, aid interpretation of biomarkers and inform therapeutic interventions targeting the removal of tau.

## Materials and methods

### Study design and participants

Patients with a neuropathological diagnosis of PSP were identified from the Queen Square Brain Bank for Neurological Disorders (QSBB) between 2010 and 2022. Consecutive patients were included, except where medical records did not contain adequate documentation of symptom onset, clinical features and disease progression. QSBB protocols and this study have been approved by the NHS Health Research Authority Ethics Committee London–Central (REC reference 23/LO/0044). All donors included in this study or their next of kin gave written informed consent.

### Clinical assessment

All patients were regularly assessed by hospital specialists in the UK throughout the course of their illness. Patients’ primary and secondary care medical records were reviewed by neurologists with experience in movement disorders. We recorded demographic information, clinical features and response to levodopa as previously described (Supplementary material).^16^ The time from symptom onset to each clinical variable was calculated, and clinical predominance type (phenotype) was assigned based on the clinical features present within the first 3 years from symptom onset according to the Movement Disorder Society (MDS) PSP criteria^17^ and a modified version of the Multiple Allocations eXtinction (MAX) rules.^18^ The first MAX rule was omitted, thus favouring the predominance type rather than certainty level when assigning the phenotype to avoid overestimating PSP-RS, which is a criticism of this system.^19,20^ To evaluate disease progression, we recorded the time from symptom onset to specific disease milestones representing different domains of functional impairment. Milestones were chosen based on previous studies^13,21^ and on the basis that they are likely to require medical attention and would be well documented in the medical records including: frequent falls and wheelchair dependence as markers of motor impairment; unintelligible speech and the insertion or recommendation of percutaneous endoscopic gastrostomy (PEG) tube placement for feeding indicating severe bulbar dysfunction; dementia; and placement in residential or nursing home care or requirement of care for most activities of daily living in the home reflecting global disability. The time from the onset of symptoms to death (survival) was also recorded. If a clinical feature was not recorded in the medical files, it was assumed absent. Ambiguity around the timing of clinical variables was resolved through consensus between raters.

### Genetics

DNA was extracted from cerebellar or frontal lobe tissue and genotyped using Illumina NeuroBooster or NeuroChip arrays.^22,23^ Genotype data were filtered using PLINK v.2;^24^ individuals and single nucleotide polymorphisms (SNPs) with >5% missing data were excluded using –mind 0.05, –geno 0.05, –maf 0.01 and hwe1e-6 filters. Genotype data were imputed against the TOPMed r2 panel^25^ with Eagle v.2.4^26^ phasing on the TOPMed Imputation Server using Minimac4. Variants previously reported to influence PSP tau pathology burden, clinical phenotype or progression were extracted from the imputed data, including *MAPT* haplotype (defined using rs1800547),^27,28^ the *TRIM11* SNP rs564309^29,30^ and the *SLC2A13* SNP rs2242367.^31^ Patients homozygous (A/A) or heterozygous (C/A) for the *TRIM11* SNP rs564309 were designated *TRIM11* (A). Similarly, patients homozygous (A/A) or heterozygous (G/A) for the *SLC2A13* SNP rs2242367 were designated *SLC2A13* (A). Patients carrying one or two copies of the *MAPT* H2 haplotype (H1/H2 or H2/H2) were classified as *MAPT* H2. We also included *APOE* genotype (defined using rs429358 and rs7412), given its association with Alzheimer’s disease (AD) pathology^32^ and PSP risk.^33^ Other genes associated with the risk of PSP were not included in this study.^34^

### Neuropathological examination

Pathological examination was performed following QSBB protocols. For histological analysis, 7 µm sections were cut from formalin-fixed, paraffin-embedded tissue blocks from representative brain regions. These included the anterior frontal lobe, encompassing the superior and middle frontal gyri; the posterior frontal lobe, containing the primary motor cortex; the temporal lobe, including the superior and middle temporal gyri; the parietal lobe, including the superior and inferior parietal lobules; the occipital lobe, containing the striate cortex; the hippocampus, sampled at the level of the lateral geniculate nucleus; the basal ganglia, including the caudate, putamen and globus pallidus; the thalamus, and subthalamic nucleus; the midbrain, at the level of the red nucleus; and the cerebellum, at the level of the dentate nucleus. Immunohistochemical staining for α-synuclein (MA1-90342; Thermo Scientific; 1:1500), amyloid-β (M0872; Dako; 1:100), phosphorylated tau (MN1020; AT8 clone; Thermo Scientific; 1:1200) and non-phosphorylated TAR DNA-binding protein 43 (TDP-43) (2E2-D3, H00023435-M01, Abnova, 1:500) was performed using automated staining platforms from Menarini Diagnostics or Ventana (Roche). Biotinylated secondary antibodies were used with horseradish peroxidase-conjugated streptavidin complex and diaminobenzidine (DAB) as the chromogen, with haematoxylin counterstaining. Appropriate positive and negative controls were included in each staining run. Pathological diagnoses were confirmed by neuropathologists experienced in neurodegenerative diseases using relevant consensus diagnostic and staging criteria including those previously developed for PSP.^5,35-41^

### Quantitative digital neuropathology

**Figure 1:**
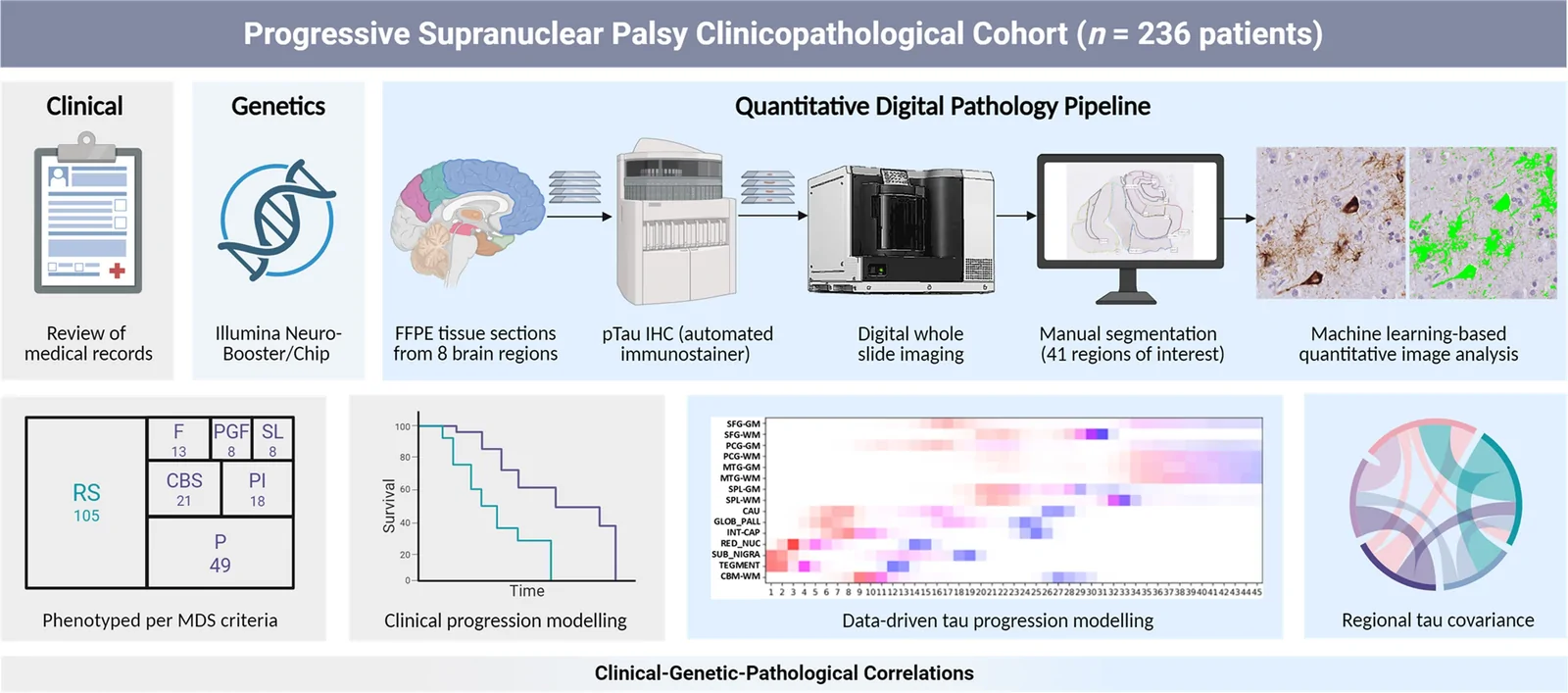
**Clinical, genetic and quantitative digital pathology pipeline.** Schematic representation of clinical, genetic and quantitative digital pathology pipeline. CBS = corticobasal syndrome; F = frontal; FFPE = formalin-fixed paraffin-embedded; IHC = immunohistochemistry; MDS = Movement Disorder Society; P = parkinsonism; PGF = pure gait freezing; PI = postural instability; pTau = phosphorylated tau; RS = Richardson’s syndrome; SL = speech/language . Created in BioRender. Cullinane, P. (2026) https://BioRender.com/c5i0tzn.

Immunohistochemistry-stained slides from eight brain regions (anterior frontal, posterior frontal, temporal and parietal lobes, hippocampus, basal ganglia, midbrain and the cerebellum) were scanned using Hamamatsu NanoZoomer S360 (26 × 76 mm slides; 40× objective; JPEG compression quality 70; pixel size 0.23 µm/pixel) or Hamamatsu NanoZoomer S60 (double 52 × 76 mm slides; 40× objective; JPEG compression quality 65; pixel size 0.22 µm/pixel) slide scanners (Fig. 1). The subthalamic nucleus, a characteristic site of PSP pathology, was excluded from quantitative analysis due to the difficulty of accurately delineating its boundaries in the context of the significant degree of atrophy commonly observed in this region. Forty-one anatomical regions of interest (ROIs) (Supplementary Table 1) were manually annotated on the digitized sections using a web-based whole-slide image (WSI) viewer (NZConnect, Hamamatsu). The annotations were exported and downloaded as .ndpa files using a custom Python script and imported into QuPath v.0.5.1^42^ for image analysis using a custom Groovy script. Stain vectors for colour deconvolution were calculated from a composite image consisting of 1000 × 1000-pixel squares derived from 270 WSIs stained for phosphorylated tau, with 1% of extreme pixels ignored. After applying the stain vectors onto each WSI, a threshold for positive phosphorylated tau staining was calculated using the Otsu^43^ automatic thresholding method via an adaptation of FIJI/ImageJ’s implementation for each brain region annotation. The automatically calculated threshold values were then used to measure the area of phosphorylated tau staining within each annotated ROI. The percentage of positive DAB staining within each brain region was calculated by: %tau = (area of phosphorylated tau staining/area of brain region annotation) × 100. We created composite values for grey matter (GM) and white matter (WM) across cortical, limbic, basal ganglia, midbrain and cerebellar regions for summary statistics (Supplementary Table 2).

### Data-driven modelling

The Subtype and Stage Inference (SuStaIn) algorithm combines progression modelling with clustering to facilitate the identification of subgroups with distinct progression patterns from cross-sectional data.^15^ Depending on the data type, subgroup progression patterns can be modelled using different approaches; in this study we used the piecewise linear *Z*-score trajectory approach for continuous data. We applied SuStaIn to quantitative tau pathology data from 15 ROIs (Supplementary Table 1), selected to include both the canonical brain regions typically affected by PSP pathology and based on quantitative tau pathology data, those regions most effective at distinguishing PSP clinical phenotypes. Neurologically healthy aged controls rarely exhibit incidental PSP tau pathology but often show incidental AD tau pathology, making them an unsuitable reference population for regional quantitative tau pathology data aimed at characterizing PSP. Therefore, to meet the SuStaIn framework’s requirement for *Z*-score normalization and in the absence of appropriate control tissue, we generated synthetic control profiles instead of using data from neurologically healthy controls. Twenty-seven synthetic control subjects were constructed to reflect minimal to absent tau pathology; values were generated for each brain region simulating 0%–5% of the true mean pathological burden with an approximately normal distribution.

The SuStaIn algorithm optimizes the assignment of individuals to subtypes and the sequence in which ROIs reach different *Z*-scores within each subtype using a data likelihood function. Model uncertainty was assessed with 1 000 000 Markov Chain Monte Carlo (MCMC) iterations, and the expectation-maximization process optimized the subtype sequences from 25 random starting points to find the maximum likelihood solution. We evaluated models comprising one to seven SuStaIn subtypes. To determine the optimal number of subtypes, we performed 10-fold cross-validation across models and calculated the cross-validation information criterion (CVIC), which evaluates the balance of model accuracy against model complexity.^15^ For computational feasibility, cross-validation was performed using 100 000 MCMC iterations per fold. We also inspected the test-set log-likelihoods plots, which assess the generalizability of the model, and the MCMC log-likelihood traces and histograms, which evaluate model fit with increasing subtypes.

### Statistical analysis

To assess differences in demographic, clinical, genetic and neuropathological features across PSP clinical phenotypes and SuStaIn subtypes we used Pearson’s chi-square or Fisher’s exact test for categorical variables, as appropriate; *P*-values were estimated using Monte Carlo simulation for sparse contingency tables. For categorical variables with more than two levels, we conducted *post hoc* pairwise Fisher’s exact tests between all phenotype pairs with Bonferroni correction for multiple comparisons. For continuous variables we used one-way ANOVA followed by pairwise comparisons with Bonferroni correction. For continuous variables that violated parametric assumptions we used the Kruskal–Wallis test with *post hoc* Wilcoxon rank-sum tests and Bonferroni correction. To assess clinical predictors of survival, we conducted an exploratory Cox proportional hazards regression analysis using the survival package in R. Demographic and genetic data, levodopa responsiveness and clinical features and milestones present within 3 years from symptom onset were included as predictors. Each predictor was examined in univariate models and in models adjusted for sex and age at onset, with phenotype included as a stratification factor. Time-varying effects were modelled using log(time) interactions where proportional hazard assumptions were violated. Generalized linear models with a gamma distribution and log link were applied to assess the relationship between tau pathology burden and survival adjusting for age at symptom onset, phenotype, sex and National Institute on Aging–Alzheimer’s Association ABC (NIA–ABC) score, a staging system that integrates regional amyloid-β pathology burden (A), Braak neurofibrillary tangle (NFT) stage (B) and cortical neuritic plaque density (C) to assess the overall severity of AD pathology.^44^ Benjamini–Hochberg correction for multiple comparisons was applied to the *P*-values for the primary predictors in both Cox regression and generalized linear models. Differences in regional tau pathology burden across PSP clinical phenotypes and SuStaIn subtypes were assessed using the Kruskal–Wallis test; where significant, this was followed by pairwise Wilcoxon rank-sum tests, with Bonferroni correction applied within each region; this was conducted both on raw values and on residuals from linear regression adjusting for age, sex and NIA–ABC score. To evaluate regional covariance in tau pathology pairwise Spearman correlations were also computed on the residuals. Strong correlations were defined as those with Bonferroni adjusted *P* < 0.05 and Spearman’s ρ > 0.5. Regional tau pathology burden was compared across sex, *MAPT* H2 haplotype, *APOE* ε4, *TRIM11* (A) and *SLC2A13* (A) status using Wilcoxon rank-sum tests, with Benjamini–Hochberg correction for multiple comparisons across regions. Bonferroni correction was applied to provide more stringent control of false positives for multiple pairwise subtype comparisons within a region, whereas the Benjamini–Hochberg procedure was used to maintain power for analyses involving a single contrast tested across many regions. The association between SuStaIn stage and both disease duration and manually assigned pathological PSP stages was performed using Spearman’s correlation. Only cases with complete data for required variables were included in analyses, except for correlation analyses, where pairwise complete observations were used. All *P*-values were two-tailed. Statistical analysis was performed using SPSS version 29 (IBM) and R (4.4.2), and graphs were generated using R. A large language model (ChatGPT, OpenAI) was used to assist in generating R code for statistical analyses and data visualization. All code, statistical tests, and outputs were manually reviewed and validated by the authors to ensure methodological appropriateness and accuracy of results.

## Results

### Demographic, clinicopathological and genetic characteristics of the cohort

We identified 241 pathologically confirmed PSP patients. Five patients were excluded due to insufficient clinical information. No pathogenic *MAPT* mutations were identified among the 193 patients with available genetic data, and no atypical histopathological features suggestive of genetic tauopathies were observed in those without sequencing data. The mean ages at symptom onset and death were 67.8 ± 7.5 and 75.8 ± 7.4 years, respectively. Thirteen patients could not be assigned a phenotype due to inadequate documentation of eye movement examination at 3 years, and one patient had a primary lateral sclerosis phenotype, which was not classifiable per MDS criteria. Demographic and clinical data for the remaining 222 patients grouped by MDS phenotype at 3 years from symptom onset are shown in Table 1. Overall, 63.6% of patients in the cohort were male, but 66.7% of PSP-corticobasal syndrome (PSP-CBS) and postural instability (PSP-PI) patients were female. PSP-frontal (PSP-F) and PSP-speech/language (PSP-SL) patients had the earliest and latest ages of symptom onset and death, respectively. Levodopa responsiveness in PSP with predominant parkinsonism (PSP-P) was twice that of PSP-RS, but sustained responsiveness beyond 2 years did not significantly differ across phenotypes. There was no significant difference in the frequency of *MAPT* H2 haplotype, *APOE* ε4, *TRIM11* (A) or *SLC2A13* (A) across clinical phenotypes. Mean ages at symptom onset and death were 6.1 [95% confidence interval (CI) 2.1, 10.1; *P* = 0.003] and 5.7 (95% CI 1.9, 9.6; *P* = 0.004) years later in *MAPT* H2 carriers compared with *MAPT* H1/H1 carriers. With the exception of more frequent Braak-NFT stages 4 and 5 pathology in patients with PSP-P compared with PSP-RS, there were no significant differences in semi-quantitative measures of AD or Lewy pathologies across PSP phenotypes as assessed by Thal phases of amyloid-β pathology, Braak-NFT staging, Consortium to Establish a Registry for Alzheimer’s Disease (CERAD) neuritic plaque scores and Braak staging for Lewy pathology (Braak-LP) (Table 1 and Supplementary Table 3). TDP-43 pathology was present in 87.5% of PSP-SL cases, considerably more than all other clinical phenotypes. Three of eight PSP-SL cases showed pathology corresponding to stage ≥ 2 limbic-predominant age-related TDP-43 encephalopathy neuropathological change (LATE-NC) and one case showed rare limbic threads consistent with mild stage 1 LATE-NC. Three additional PSP-SL cases had TDP-43 positive neuropil thread pathology in the hippocampal CA1 subregion and/or subiculum without involvement of the amygdala. Hippocampal sclerosis was absent in all PSP-SL cases with TDP-43 co-pathology.

**Table 1:** Demographic, clinical and pathological characteristics by PSP subtype

	Total (*n* = 222)	RS (*n* = 105)	*P* (*n* = 49)	CBS (*n* = 21)	PI (*n* = 18)	F (*n* = 13)	SL (*n* = 8)	PGF (*n* = 8)	*P* (adj.)^a^
Female sex	82 (36.9)	35 (33.3)	12 (24.5)	14 (66.7)	12 (66.7)	4 (30.8)	1 (12.5)	4 (50)	0.002; CBS versus P
AAO	67.8 ± 7.6	67.5 ± 6.9	68.5 ± 8.9	70.5 ± 7.9	66.8 ± 5.8	61.7 ± 7.4	71.8 ± 7.7	67.1 ± 10.7	0.024; CBS versus F
AAD	75.8 ± 7.5	73.8 ± 6.5	78.9 ± 8.0	77.9 ± 6.3	76.7 ± 6.7	70.7 ± 8.2	80.9 ± 8.2	78.8 ± 8.6	<0.001; P versus RS, F; F versus SL
Survival	8.0 ± 3.2	6.3 ± 2.1	10.4 ± 3.8	7.4 ± 2.4	9.9 ± 1.9	9 ± 2.3	9.1 ± 1.7	11.7 ± 3.8	<0.001^b^
CoD									0.162
PSP	114 (51.4)	53 (50.5)	24 (49.0)	11 (52.4)	8 (44.4)	10 (76.9)	4 (50)	4 (50)	–
Infection	91 (41.0)	46 (43.8)	20 (40.8)	9 (42.9)	10 (55.6)	2 (15.4)	3 (37.5)	1 (12.5)	–
Other	17 (7.7)	6 (5.7)	5 (10.2)	1 (4.8)	0 (0)	1 (7.7)	1 (12.5)	3 (37.5)	–
L-dopa response									0.001
Yes	53 (29.4)	21 (24.4)	25 (53.2)	1 (6.2)	1 (7.7)	2 (25.0)	1 (50)	2 (25.0)	P versus RS, CBS
Sustained	12 (6.7)	3 (3.5)	6 (12.5)	0 (0)	0 (0)	1 (12.5)	0 (0)	2 (25.0)	0.083
*APOE* ε4	38 (21.2)	21 (25.3)	8 (20)	2 (11.8)	3 (21.4)	2 (20)	1 (14.3)	1 (12.5)	0.939
*MAPT* H2	15 (8.4)	5 (6.0)	4 (10)	2 (11.8)	2 (14.3)	1 (10)	1 (14.3)	0 (0)	0.649
*TRIM11* (A)	32 (17.9)	18 (21.7)	5 (12.5)	5 (29.4)	1 (7.1)	2 (20)	0 (0)	1 (12.5)	0.503
*SLC2A13* (A)	77 (43.0)	32 (38.6)	21 (52.5)	8 (47.1)	7 (50)	3 (30)	4 (57.1)	2 (25.0)	0.577
NIA-ABC									0.099
None	70 (31.7)	32 (30.5)	14 (28.6)	10 (47.6)	2 (11.1)	5 (41.7)	2 (25.0)	5 (62.5)	–
Low	126 (57.0)	65 (61.9)	25 (51.0)	10 (47.6)	14 (77.8)	6 (50)	4 (50)	2 (25.0)	–
Int.	22 (10)	8 (7.6)	7 (14.3)	1 (4.8)	2 (11.1)	1 (8.3)	2 (25.0)	1 (12.5)	–
High	3 (1.4)	0 (0)	3 (6.1)	0 (0)	0 (0)	0 (0)	0 (0)	0 (0)	–
A-P LP									0.059
No	211 (95.5)	102 (97.1)	47 (95.9)	21 (100)	14 (77.8)	11 (91.7)	8 (100)	8 (100)	–
Yes	10 (4.5)	3 (2.9)	2 (4.1)	0 (0)	4 (22.2)	1 (8.3)	0 (0)	0 (0)	–
Braak-LP									0.682
0	195 (88.2)	95 (90.5)	40 (81.6)	17 (81.0)	18 (100)	11 (91.7)	7 (87.5)	7 (87.5)	–
1	3 (1.4)	1 (1.0)	0 (0)	1 (4.8)	0 (0)	0 (0)	1 (12.5)	0 (0)	–
2	2 (0.9)	1 (1.0)	1 (2.0)	0 (0)	0 (0)	0 (0)	0 (0)	0 (0)	–
3	4 (1.8)	2 (1.9)	1 (2.0)	1 (4.8)	0 (0)	0 (0)	0 (0)	0 (0)	–
4	8 (3.6)	3 (2.9)	3 (6.1)	0 (0)	0 (0)	1 (8.3)	0 (0)	1 (12.5)	–
5	4 (1.8)	2 (1.9)	1 (2.0)	1 (4.8)	0 (0)	0 (0)	0 (0)	0 (0)	–
6	5 (2.3)	1 (1.0)	3 (6.1)	1 (4.8)	0 (0)	0 (0)	0 (0)	0 (0)	–
TDP-43									<0.001
None	197 (89.1)	99 (94.3)	42 (85.7)	20 (95.2)	16 (88.9)	11 (91.7)	1 (12.5)	8 (100)	SL versus RS, P, PGF, PI, CBS, F
L-NC 1	1 (0.5)	0 (0.0)	0 (0.0)	0 (0.0)	0 (0.0)	0 (0.0)	1 (12.5)	0 (0.0)	
L-NC ≥ 2	16 (7.2)	4 (3.8)	7 (14.3)	1 (4.8)	0 (0.0)	1 (8.3)	3 (37.5)	0 (0.0)	
NOS	7 (3.2)	2 (1.9)	0 (0.0)	0 (0.0)	2 (11.1)	0 (0.0)	3 (37.5)	0 (0.0)	

Data shown as number of patients (percentage) for categorical variables and mean number of years (standard deviation) for continuous variables. Percentages are calculated among patients with available (non-missing) data for L-dopa response and genotype data within each phenotype. AAO = age at onset; AAD = age at death; A-P = amygdala predominant; CBS = corticobasal syndrome; CoD = cause of death; F = frontal; Int. = intermediate; L-NC = limbic-predominant age-related TDP-43 encephalopathy-neuropathological change; LP = Lewy pathology; NIA–ABC = National Institute on Aging–Alzheimer’s Association ABC score; NOS = not otherwise specified (refers to cases with TDP-43 positive neuropil thread pathology in the CA1 subregion of the hippocampus and/or subiculum without involvement of the amygdala); P = parkinsonism; PGF = pure gait freezing; PI = postural instability; PSP = progressive supranuclear palsy; RS = Richardson's syndrome; SL = speech/language; sustained = sustained L-dopa response >2 years.

^a^Adjusted significant pairwise comparisons.

**Figure 2:**
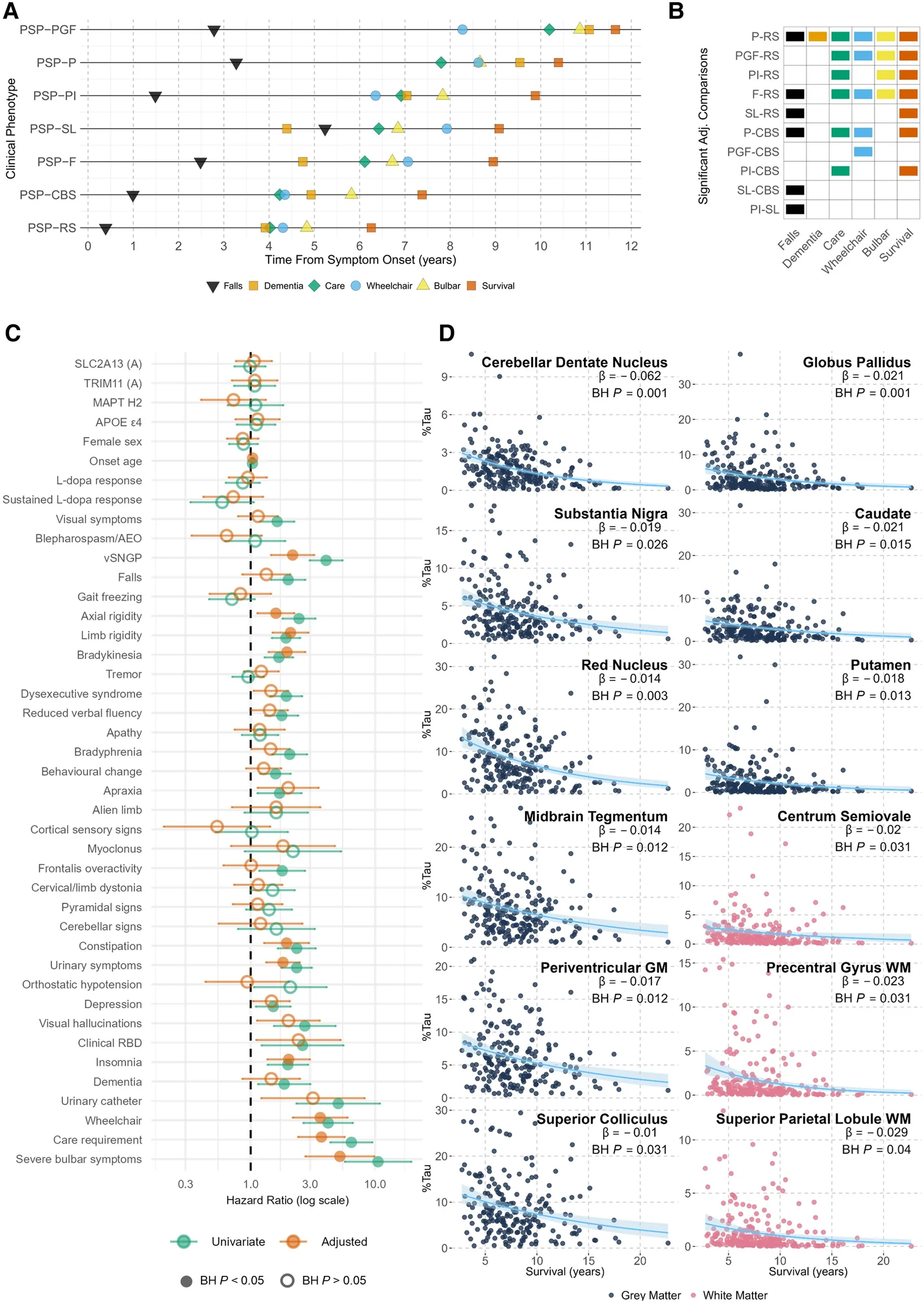
**Clinical milestones by phenotype, clinical predictors of survival and association between regional tau pathology and survival**. (**A**) The line plot shows the mean time from symptom onset to key clinical milestones and survival for each PSP phenotype. (**B**) The tile plot displays significant pairwise differences in milestone timing and survival between phenotypes; coloured tiles indicate milestones with significant differences for each phenotype comparison (Bonferroni-corrected *P* < 0.05). (**C**) The forest plot shows hazard ratios (HRs) and 95% confidence intervals for overall survival from univariate (green) and adjusted (orange) Cox regression models. Filled symbols indicate Benjamini–Hochberg-corrected (BH) *P* < 0.05. Adjusted models included sex and age at onset as covariates and were stratified by phenotype. (**D**) Scatter plots show the relationship between tau pathology burden (%Tau) in individual brain regions and survival adjusting for onset age, phenotype, sex and National Institute on Aging (NIA)-ABC score, with each point representing an individual patient. Each panel displays a fitted gamma regression line with 95% confidence interval shading. The regions shown were significant after Benjamini–Hochberg correction. β coefficients and adjusted *P*-values are annotated for each model. Visual symptoms: blurred vision, diplopia or photophobia; AEO = apraxia of eyelid opening; CBS = corticobasal syndrome; F = frontal; GM = grey matter; P = parkinsonism; PGF = pure gait freezing; PI = postural instability; PSP = progressive supranuclear palsy; RS = Richardson's syndrome;; RBD = REM sleep behaviour disorder; SL = speech/language; vSNGP = vertical supranuclear gaze palsy; WM = white matter.

^b^Adjusted significant pairwise comparisons for survival are shown in Fig. 2B.

### Clinical and pathological predictors of survival

Overall, the mean survival from symptom onset was 8 ± 3.2 years. Mean survival and latencies to clinical milestones between PSP phenotypes are shown in Fig. 2A, and significant differences in the timing of clinical milestones and survival between phenotypes are shown in Fig. 2B. Cause of death did not differ by phenotype (Table 1). After adjusting for onset age, phenotype and sex, several clinical features and milestones present within the first 3 years from symptom onset predicted shorter survival, including vertical supranuclear gaze palsy, axial and limb rigidity, bradykinesia, constipation, urinary symptoms, insomnia, wheelchair use, the requirement for nursing or home care and severe bulbar symptoms (Fig. 2C and Supplementary Table 4). Several other features were nominally significant including dysexecutive symptoms, reduced verbal fluency, bradyphrenia, apraxia, visual hallucinations, clinical rapid eye movement sleep behaviour disorder (RBD), depression and urinary catheterization. There was no significant difference in the frequency of Lewy pathology in the amygdala or canonical Braak-LP regions among patients who experienced constipation, RBD or visual hallucinations within 3 years. Several other features were also associated with reduced survival in univariate analyses. Regional quantitative tau pathology data were available for 235 cases; missing data for individual regions are detailed in Supplementary Table 1. Adjusting for onset age, phenotype, sex and NIA–ABC score, survival decreased with increasing tau pathology burden in the cerebellar dentate nucleus, several midbrain and basal ganglia GM regions, the centrum semiovale, and precentral gyrus and superior parietal lobule WM (Fig. 2D and Supplementary Table 5).

### Clinical and genetic determinants of regional tau pathology

**Figure 3:**
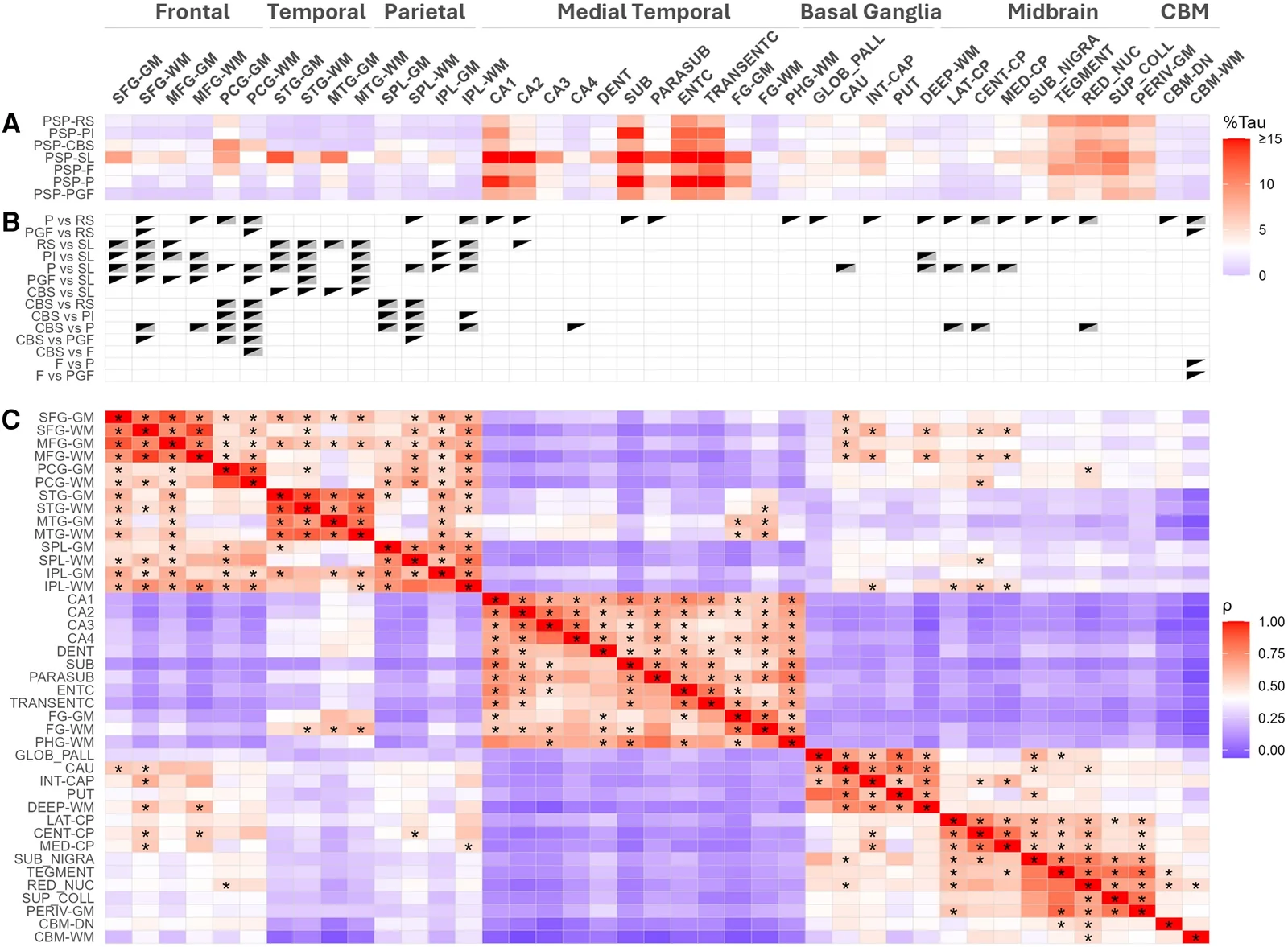
**Regional tau pathology burden by clinical phenotype and regional covariance.** (**A**) The heat map shows mean tau pathology burden (%Tau) in 41 individual brain regions for each clinical phenotype. (**B**) The tile plot shows brain regions where there are significant pairwise differences in tau pathology burden between phenotypes; within each tile, a black upper triangle indicates a significant pairwise difference (*P *< 0.05, Bonferroni-corrected) in the unadjusted analysis and a grey lower triangle indicates pairwise comparisons that remain significant in the analysis adjusted for age, sex and National Institute on Aging–Alzheimer’s Association ABC (NIA–ABC) score. (**C**) The correlation heat map shows pairwise Spearman correlations between residualized tau pathology values adjusted for age, sex and NIA–ABC score; asterisks mark correlations with ρ > 0.5 that were significant after Bonferroni correction for multiple testing. CBM = cerebellum; CBS = corticobasal syndrome; F = frontal; GM = grey matter; P = parkinsonism; PGF = pure gait freezing; PI = postural instability; PSP = progressive supranuclear palsy; RS = Richardson's syndrome; SL = speech/language; WM = white matter.

**Figure 4:**
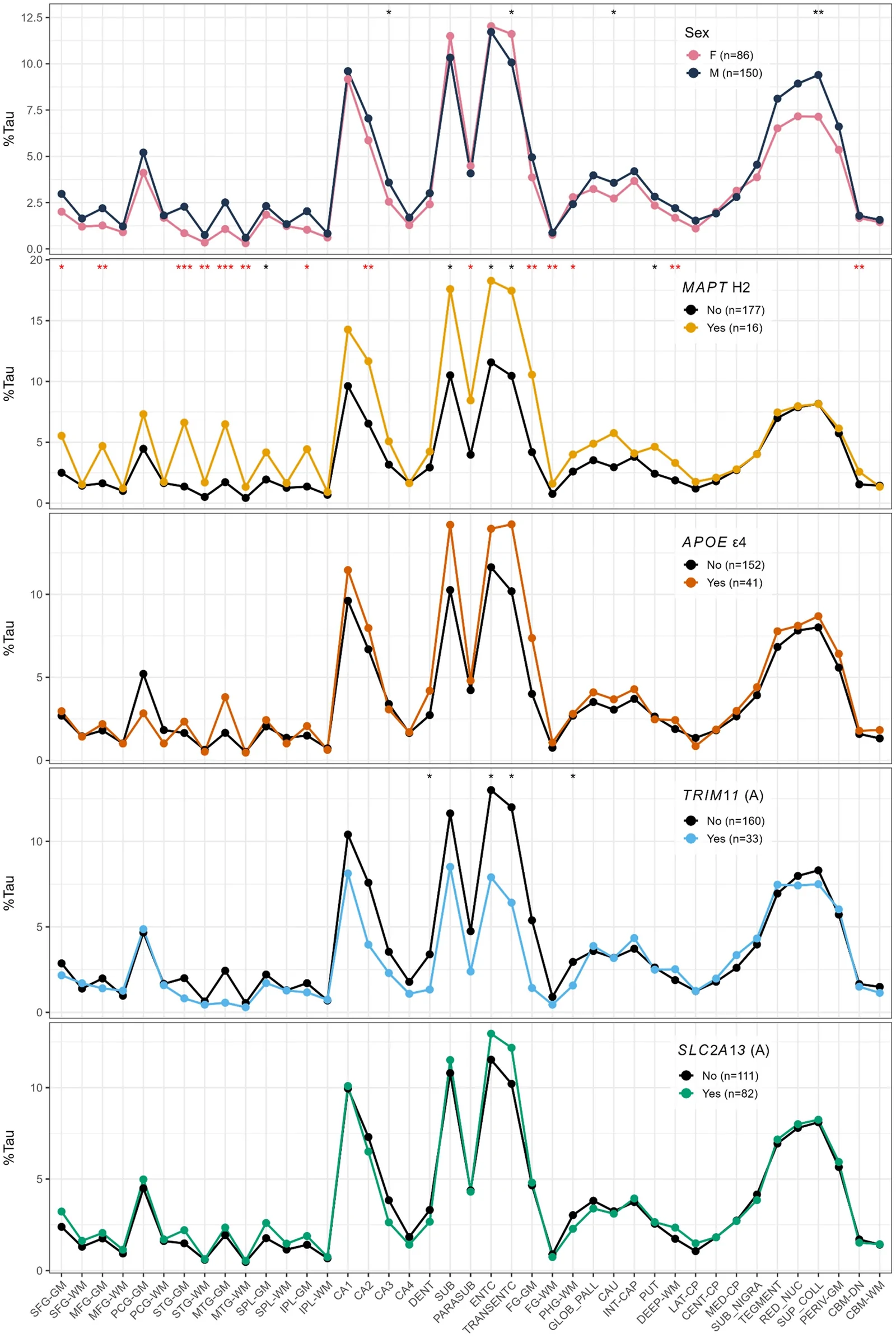
**Regional tau pathology by sex and genotype.** The line plots show mean tau pathology burden (%Tau) across 41 brain regions for each category of sex, *MAPT* H2, *APOE* ε4, *TRIM11* (A) and *SLC2A13* (A) in all progressive supranuclear palsy cases. Error bars have been omitted for clarity. Asterisks indicate nominally significant differences between groups in individual regions (*P < 0.05, **P < 0.01, ***P < 0.001). Red significance symbols denote regions that remain significant following Benjamini–Hochberg correction for multiple comparisons. Sample sizes (*n*) for each group are indicated in the legends. F = female; M = male.

We identified anatomically congruent differences in quantitative tau pathology burden between clinical phenotypes, including higher GM and WM pathology in the superior and middle frontal and temporal gyri of PSP-SL patients, the precentral gyrus and superior parietal lobule of PSP-CBS, and in the precentral gyrus and several subcortical regions of PSP-RS compared with PSP-P (Fig. 3A and B and Supplementary Table 6). Male patients had nominally higher tau pathology in the superior colliculus, caudate and hippocampal CA3 subregion, although these differences were not significant after correcting for multiple comparisons (Fig. 4). Restricting the analysis to PSP-RS patients, tau pathology in males was even greater in the superior colliculus, with additional nominal differences in the red nucleus, midbrain tegmentum and periventricular GM (Supplementary Fig. 1). In patients with PSP-CBS, males had nominally higher tau pathology in precentral gyrus GM and WM. *MAPT* H2 haplotype carriers had significantly higher tau pathology than H1/H1 carriers in several cortical and limbic regions, centrum semiovale and cerebellar dentate nucleus (Fig. 4). *TRIM11* (A) carriers had nominally lower tau pathology in the hippocampal dentate gyrus, entorhinal and transentorhinal cortices and parahippocampal gyrus WM. After excluding patients with intermediate or high AD co-pathology from these analyses, differences in cortical grey matter reduced in magnitude and previously significant differences in the subiculum, parasubiculum, transentorhinal cortex and entorhinal cortex were lost in the *MAPT* haplotype comparisons. Similarly, nominally significant differences in tau pathology within limbic regions were lost in the *TRIM11 (A)* comparisons (Supplementary Fig. 2). Regional tau pathology did not vary by *APOE* ε4 or *SLC2A13* (A) status (Fig. 4). After adjusting for sex, phenotype and NIA–ABC score, older age at symptom onset was associated with greater tau pathology in limbic GM and WM, and with lower pathology in midbrain GM and the deep WM composite. Similarly, older age at death corresponded to greater tau pathology in limbic regions and reduced tau pathology burden in midbrain GM, deep WM composite and cerebellar WM (Supplementary Table 7).

### Regional covariance in quantitative tau pathology

In general, tau pathology in adjacent ROIs correlated strongly with each other (Fig. 3C). Significant correlations in tau pathology burden were also observed between ROIs in anatomically connected but spatially distributed brain regions, including between the superior and middle frontal gyri WM, centrum semiovale, internal capsule and cerebral peduncle. There were also correlations between superior frontal gyrus GM and WM pathology and that of the caudate nucleus, while precentral gyrus GM tau pathology was correlated with that in the red nucleus. Tau pathology load in both the substantia nigra and red nucleus correlated with that in the caudate. Cerebellar dentate nucleus and WM tau pathology burden also correlated with that in the red nucleus.

### Spatiotemporal trajectories of PSP tau pathology progression

**Figure 5:**
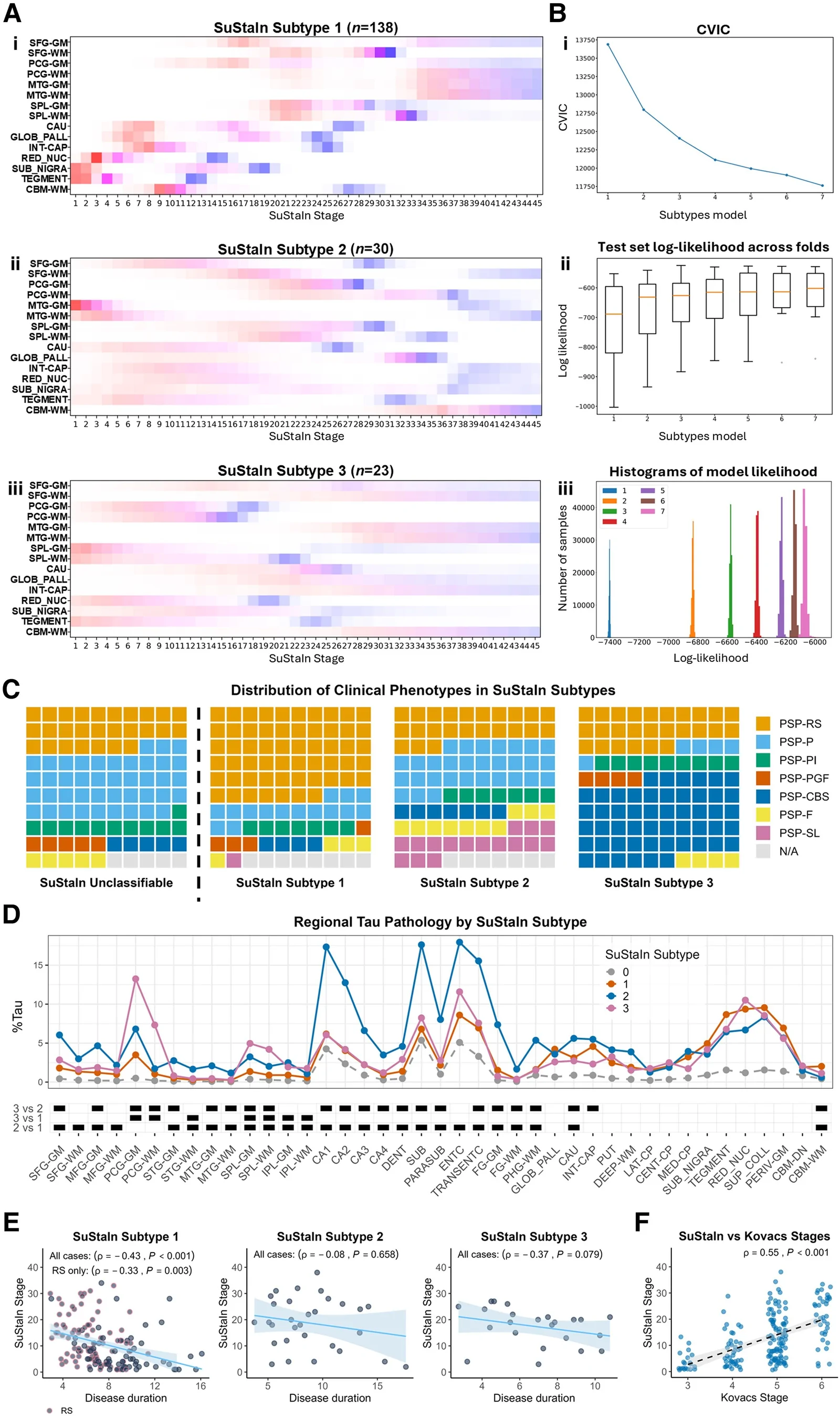
**Subtype and stage inference modelling of PSP pathology.** (**A**) Positional variance diagrams show the progression patterns of regional tau pathology inferred by the SuStaIn model for each of the three data-driven subtypes (**i**–**iii**). Each row corresponds to a specific brain region, and each column to a SuStaIn stage; colour intensity reflects the probability that a given brain region becomes affected at a specific stage, with red indicating higher probability, blue lower probability and white indicating low or no probability. (**B**) The line plot shows cross-validated information criterion (CVIC) scores for SuStaIn models with increasing numbers of subtypes; lower CVIC values indicate better model fit (**i**). The boxplots of test-set log-likelihoods across folds reflect model generalizability (**ii**). The histogram displays model likelihoods, summarizing the distribution of likelihood values across the dataset (**iii**). (**C**) The waffle plots show the proportional distribution of PSP clinical phenotypes within each SuStaIn subtype and unclassified cases, with each square representing 1% of the corresponding subtype. (**D**) The lower line plot displays mean regional tau pathology burden across individual brain regions for SuStaIn subtypes 1–3 and unclassifiable cases (subtype 0), and the corresponding tile plot indicates regions with significant pairwise differences in tau pathology burden; black tiles indicate regions with Bonferroni adjusted *P* < 0.05. (**E**) The scatter plots show the relationship between SuStaIn stage and survival across SuStaIn subtypes 1–3; points represent individual cases, with fitted regression lines (blue) and 95% confidence intervals (shaded). In subtype 1, PSP-RS cases are highlighted with a coral outline. Correlation coefficients (*ρ*) and associated *P*-values are annotated. (**F**) The final panel compares SuStaIn stages derived from machine learning-based quantitative tau burden data with Kovacs’ stages based on semi-quantitative assessment of tau pathology, with regression line (black dashed line) and 95% confidence interval (shaded) shown; the correlation coefficient (*ρ*) and associated *P*-value are annotated. CBS = corticobasal syndrome; F = frontal; P = parkinsonism; PGF = pure gait freezing; PI = postural instability; PSP = progressive supranuclear palsy; RS = Richardson's syndrome; SL = speech/language.

Applying SuStaIn to quantitative tau pathology data from all PSP patients with NIA–ABC scores ≤1 yielded three subtypes (Fig. 5A). Cases with intermediate or high Alzheimer’s neuropathological change (NIA–ABC ≥ 2) were excluded to minimize the effect of AD co-pathology. Although the CVIC reached its minimum value in the seven-subtype model (Fig. 5B) indicating improved fit with up to seven subtypes, the test-set log-likelihoods plateaued after three subtypes (Fig. 5B), indicating limited generalizability beyond three subtypes. The choice of three subtypes was further supported by inspection of the positional variance diagrams (PVDs), which showed low positional uncertainty and distinct, anatomically plausible progression patterns in the three-subtype model (Fig. 5A). In contrast, additional subtype models introduced more fragmented or overlapping trajectories with increased uncertainty. The MCMC log-likelihood traces and histograms show separation with up to five subtypes (Fig. 5B), indicating that further subtypes may be identified in larger cohorts.

**Figure 6:**
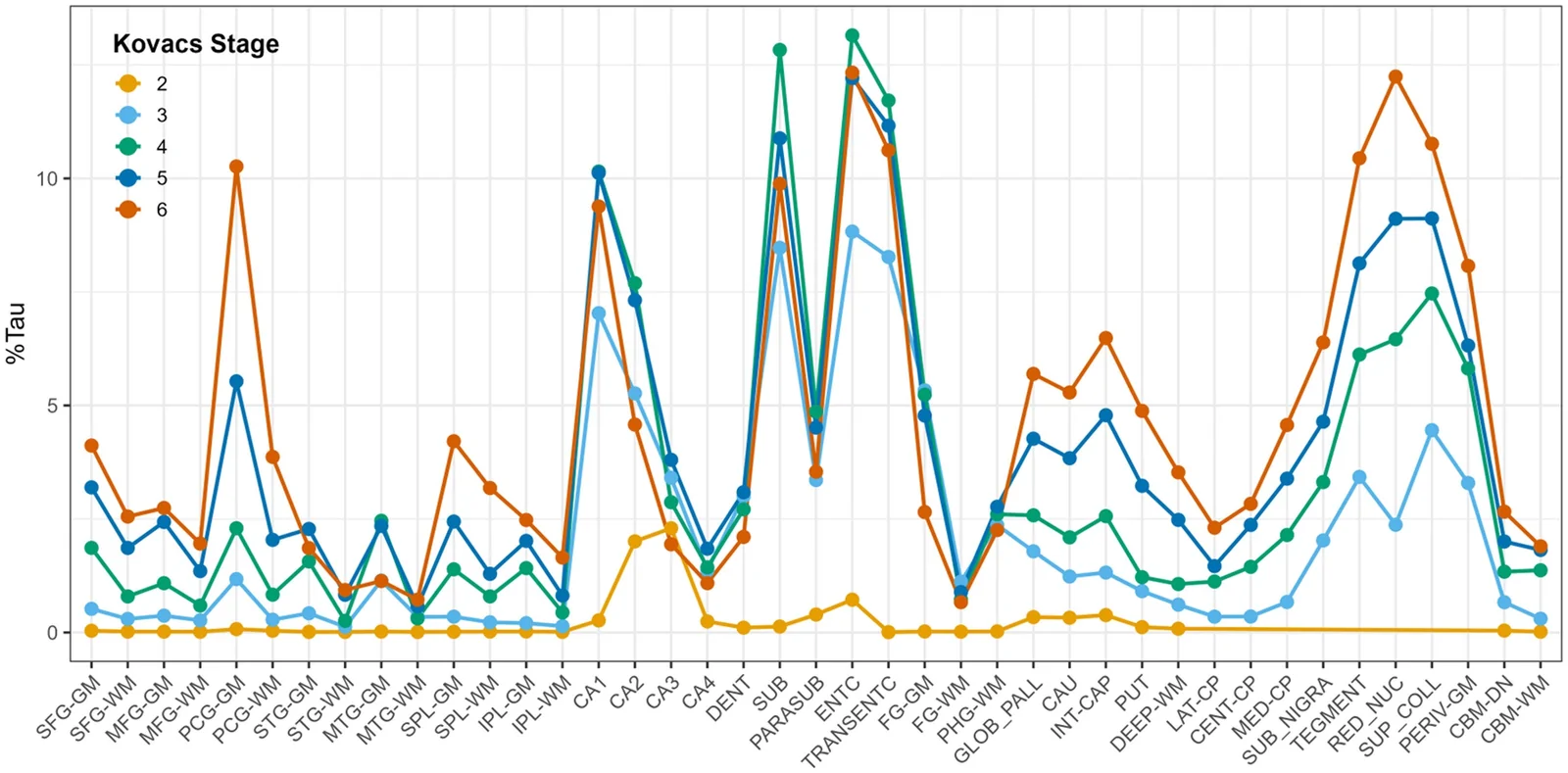
**Mean regional tau pathology burden across semi-quantitatively defined PSP stages.** Each line shows mean quantitative tau pathology burden (%Tau) in 41 brain regions across each semi-quantitatively scored PSP stage. PSP = progressive supranuclear palsy.

SuStaIn-defined subtype 1 shows progression from subcortical GM caudally to cerebellar WM and then rostrally to precentral gyrus GM, followed by involvement of other cortical regions (Fig. 5A). Subtype 2 and subtype 3 show simultaneous early involvement of subcortical GM and neocortical regions. Subtype 2 is distinguished by early involvement of middle temporal gyrus GM and WM, followed by anterior superior frontal gyrus GM. In subtype 3 the emphasis is more posterior in precentral gyrus and superior parietal lobule GM and WM. PSP-RS, PSP-PI, PSP-P and PSP with pure gait freezing (PSP-PGF) combined comprised 83.2% of subtype 1 patients compared with 60% and 43.4% of subtype 2 and subtype 3, respectively (Fig. 5C). Conversely, cortical phenotypes (PSP-CBS, PSP-SL and PSP-F) comprised a considerably greater proportion of subtype 2 (33.4%) and subtype 3 patients (56.5%) compared with subtype 1 (8.6%). Specifically, 85.9% of patients classified as RS, 67.7% of PSP-P and 71.4% of PSP-PI patients were included in subtype 1, whilst 65.7% of those with either PSP-CBS, PSP-SL or PSP-F were categorized in subtype 2 or 3. There were no significant differences in sex, genetic factors or age at symptom onset between SuStaIn subtypes (Supplementary Table 8). Mean disease duration was 2 years longer in subtype 2 compared with subtype 3, and subtype 2 patients were, on average, 3.4 years older at death than subtype 1 patients. Compared with subtype 1, tau pathology was higher in several regions of subtype 2 patients including the superior and middle frontal and temporal gyri WM and GM, centrum semiovale and the caudate nucleus, and lower in cerebellar WM (Fig. 5D). Tau pathology was also greater in several medial temporal structures of subtype 2 cases suggestive of low level AD or primary age-related tauopathy. Tau pathology was greater in the precentral gyrus and superior and inferior parietal lobule GM and WM in subtype 3 (Fig. 5D). Nineteen patients were not classifiable by SuStaIn (subtype 0) and the mean tau pathology load was considerably lower in all regions of these patients. Almost half of the unclassifiable patients had a PSP-P phenotype (*n* = 8), followed by PSP-RS (*n* = 5), PSP-PI (*n* = 2), PSP-CBS (*n* = 1), PSP-F (*n* = 1), PSP-PGF (*n* = 1) and no MDS phenotype assigned (*n* = 1). Increasing SuStaIn stage was associated with shorter survival in subtype 1 (Fig. 5E). There were no significant correlations between SuStaIn stage and survival in subtypes 2–3. Semi-quantitative staging of tau pathology as described by Kovacs^5^ corresponded well to both SuStaIn stage (Fig. 5F) and quantitative measurements of regional tau pathology (Fig. 6).

## Discussion

Using standardized clinical criteria and automated digital quantitative pathology methods, this comprehensive clinicopathological study has elucidated: (i) the longitudinal clinical course and clinical features that predict survival in a large cohort of patients with PSP; (ii) region-specific differences in tau pathology burden associated with phenotypic diversity and clinical progression; (iii) potential contributions of sex, genotype and co-pathology towards clinical phenotype and regional tau pathology burden; and (iv) spatiotemporal patterns of tau pathology covariance and progression.

The overall survival observed in our cohort was consistent with previous studies reporting mean disease durations of 7–8 years for PSP overall and 5–7 years for PSP-RS.^12,13,21,45-49^ Comparing non-RS phenotype-specific survival across studies is more challenging because of differences in clinical diagnostic criteria before 2017, compounded by the fact that PSP clinical phenotype is not necessarily stable over time.^50^ Also consistent with previous clinicopathological studies, features associated with reduced survival include older age at symptom onset,^21,49,51-53^ early constipation and urinary symptoms,^54^ supranuclear gaze palsy,^55-57^ severe bulbar dysfunction^47,48,51,52^ and nominally, cognitive symptoms.^48,52,53,58^ Our results also support the previous clinical observation that sleep disorders may predict shorter survival in PSP.^59^

We identified several phenotype-specific regional tau pathology signatures that support and extend prior findings that clinical phenotype corresponds to the neuroanatomical distribution of tau pathology,^5-9,60^ although direct comparisons are again challenging because of non-standardized clinical phenotyping and the practice of combining phenotypes.^61^ As anticipated from previous studies,^5,9,62^ PSP-RS patients exhibited significantly higher tau pathology across several neocortical, basal ganglia, midbrain and cerebellar structures compared with PSP-P, and PSP-PGF patients had the lowest tau pathology across most neocortical regions and the cerebellum. Neocortical tau pathology burden in PSP-CBS patients was greatest in the precentral gyrus and superior parietal lobule, while the heaviest pathology in PSP-SL was in the superior and middle temporal gyri GM. However, precentral gyrus WM rather than GM was most discriminatory for PSP-CBS, while tau pathology load in middle and superior temporal WM was more specific for PSP-SL than corresponding GM pathology burden. This distribution of pathology is consistent with white matter tract degeneration identified using diffusion weighted MRI in patients with PSP-SL.^63^ As in previous clinicopathological work using MDS clinical criteria,^5^ PSP-F patients did not have a distinct cortical tau pathology signature, which may reflect limitations in the anatomical regions of the frontal lobe available for analysis, the specificity of clinical criteria, or pathomechanisms relating to network disruption.

Regarding regional tau pathology and clinical progression, we identified 12 specific brain regions where tau pathology burden is associated with survival, including the dentate nucleus, substantia nigra, red nucleus and caudate as previously identified in smaller studies,^9,14,64^ as well as several additional associations including three WM regions. While the current study focused on total tau pathology burden, there may also be cell-type-specific contributions to survival with previous work comparing pathological features in long- and short-duration PSP patients indicating that disease progression may be more strongly influenced by oligodendroglial rather than neuronal tau pathology.^57^

Although previous studies suggest that co-pathologies do not influence PSP phenotype or progression,^12,13^ we observed that despite being infrequent overall, limbic TDP-43 co-pathology was present in most PSP-SL patients. This observation may reflect shared molecular pathways, and previous work in corticobasal degeneration has implicated TDP-43 co-pathology in modifying clinical phenotype.^65^ In our cohort, PSP-SL cases were, on average, 5.1 years older than the mean cohort age at death, suggesting that TDP-43 co-pathology in these patients may alternatively be ageing related. Data regarding sex-related differences in PSP have been inconsistent,^66^ with some studies indicating that male patients have shorter survival^21,49^ and that female patients have worse executive dysfunction and faster cognitive decline.^67^ While no sex-related differences in clinical progression were observed, the predominance of female patients in the PSP-CBS group may indicate differences in selective regional vulnerability, supported by nominally lower precentral gyrus tau pathology in female patients with PSP-CBS, implying that they may develop associated clinical symptoms despite less overall tau accumulation. In line with prior research, we did not find a significant association between *MAPT* haplotype or *APOE* genotype and survival,^28,68^ nor did we observe a significant association between the presence of the *SLC2A13* SNP rs2242367 and survival in our models.^31^ In contrast with a previous report showing reduced tau pathology among *MAPT* H2 carriers^27^ we found higher tau pathology burden in several cortical and subcortical regions in these patients. However, *MAPT* H2 carriers were older at age of symptom onset and the differences in cortical and limbic tau pathology diminished after excluding those with NIA–ABC scores ≥2 suggesting that this observation may, at least in part, be ageing related. Mildly increased overall neurofibrillary tangle severity has previously been reported in PSP patients carrying the *TRIM11* rs564309 minor allele (A); this effect was not region specific, and no differences were observed for other tau inclusions, including tufted astrocytes and coiled bodies.^30^ In the present study, *TRIM11* (A) patients had nominally lower tau pathology burden in several limbic regions, but none remained significant after correction for multiple comparisons.

Work combining resting-state functional MRI connectomics with ^18^F-PI-2620 tau-PET and post-mortem tau pathology data suggests that brain connectivity underlies stronger inter-regional correlations in pathology burden measured both *in vivo* and post-mortem.^69^ Significant correlations in tau pathology were observed between anatomically connected brain regions in the present study, providing further evidence for inter-regional connectivity in the spreading of tau pathology. Strong correlations between tau pathology burden in distant brain regions may indicate spread via long-range white matter tracts (white matter of superior and middle frontal gyri, centrum semiovale, internal capsule and cerebral peduncle) including frontostriatal projections (superior frontal cortex to caudate), as well as more localized cortico-cortical association fibres and the nigrostriatal pathway (substantia nigra to caudate). Strong correlations between tau pathology burden in the precentral gyrus GM and the red nucleus, as well as between the red nucleus and the dentate nucleus, may support tau pathology spread along corticorubral and dentatorubral pathways, respectively. These data could also be interpreted that functionally or structurally connected brain regions share intrinsic vulnerability factors as indicated by work combining AV-1451 tau PET imaging and resting state functional MRI showing that increased metabolic demand and a lack of trophic support are responsible for tau accumulation in PSP, rather than strong functional connectivity.^70^

The SuStaIn algorithm reconstructs progression patterns from cross-sectional data and has been applied to brain MRI, fluorodeoxyglucose (FDG)-PET and ^18^F-Florzolotau-PET data from PSP patients, each identifying two subtypes.^11,71,72^ MRI-SuStaIn identified a subcortical subtype with early brainstem, superior cerebellar peduncle and dentate nucleus atrophy, and a cortical subtype with early frontal and insular atrophy alongside brainstem atrophy.^71^ Approximately 80% of patients with PSP-P, PSP-PGF and PSP-RS clinical phenotypes were assigned to the MRI-SuStaIn subcortical subtype, and a similar proportion of patients with cortical phenotypes (PSP-CBS, PSP-SL, PSP-F) were assigned to the MRI-SuStaIn cortical subtype.^71^ FDG-PET-SuStaIn also identified a brainstem subtype with initial midbrain hypometabolism before progression to striatal and cortical structures, and a cortical subtype with early prefrontal hypometabolism preceding that in the caudate and midbrain structures.^72^ In contrast to MRI-SuStaIn, most of the patients with PSP-RS or PSP-P were assigned to the cortical FDG-PET-SuStaIn subtype.^72^  ^18^F-Florzolotau-PET-SuStaIn revealed one subtype progressing from subcortical (red nucleus, subthalamic nucleus, raphe nucleus and globus pallidus) to cortical regions and another subtype with early simultaneous involvement of both subcortical and cortical brain regions.^11^ In this study patients were classified as having either PSP-RS or non-RS phenotypes, with 92% of the latter group comprising PSP-P and PSP-PGF patients; 61% of PSP-RS and 71% of non-RS patients were assigned to the early subcortical SuStaIn subtype.^11^

Our findings also provide evidence for different spatiotemporal patterns of PSP tau progression wherein most patients have early midbrain and basal ganglia involvement followed by cerebellar and subsequent cortical involvement (subtype 1), whilst others have early simultaneous involvement of both cortical and subcortical regions (subtypes 2 and 3). The large proportion of cases with non-RS clinical phenotypes in the present study, along with granular quantitative tau pathology data, enabled the separation of patients with early simultaneous involvement of subcortical and cortical regions into those with more prominent temporal, followed by anterior frontal (subtype 2) or posterior frontal and parietal (subtype 3) involvement. The progression pattern in SuStaIn subtype 1 is consistent with the current pathological staging scheme for PSP,^5^ but the additional subtypes observed indicate that alternative progression patterns may occur, extending the current staging framework. These alternative patterns suggest that some PSP cases show early neocortical vulnerability rather than primarily subcortical involvement with later cortical spread. Early cortical involvement may underlie PSP-CBS, PSP-SL and PSP-F phenotypes, consistent with their enrichment in SuStaIn subtypes 2 and 3 and may reflect differing mechanisms with implications for therapeutic strategy. The distribution of clinical phenotypes in SuStaIn subtype 1, identified using quantitative tau pathology data, broadly corresponds with the MRI-SuStaIn subcortical subtype,^71^ thus supporting a common progression pattern for most patients with PSP-RS and PSP-P. The agreement is weaker for the cortical subtype, indicating that factors beyond regional tau pathology burden, including disrupted functional connectivity^73^ or intrinsic cellular susceptibility may contribute to spatiotemporal atrophy patterns in these patients. However, this discrepancy may also reflect potential confounding by age-related tau pathology in the present study, differences in anatomical regions used for SuStaIn across studies and the possible inclusion of patients with non-PSP pathology in imaging studies.

This work shares limitations common to post-mortem brain bank studies, including the potential for referral bias, non-standardized clinical assessments performed by different professionals and possible under-ascertainment from assuming undocumented clinical features were absent. To limit this potential bias, only cases with detailed information were included. Autonomic function testing and polysomnography were not available, and we recorded all visual hallucinations irrespective of duration, or potential precipitating factors. A further methodological constraint is that the quantitative digital pathology approach used in this study cannot distinguish PSP tau pathology from that due to AD or other tau co-pathologies, including argyrophilic grain disease and ageing-related tau astrogliopathy. To minimize the influence of AD co-pathology, we adjusted for NIA–ABC scores or excluded patients with NIA–ABC scores ≥2 in the clinicopathological and data-driven analyses. However, AT8-positive neuritic plaques may still contribute to cortical and hippocampal tau burden and cannot be fully disentangled from PSP tau pathology with the methods used in this study, although the associations observed between tau burden and clinical phenotype in subcortical white matter—a region that does not contain neuritic plaques—suggest that our main clinicopathological findings are unlikely to be strongly influenced by plaque-associated tau. Of note, we used non-phosphorylated TDP-43 immunohistochemistry to screen for TDP-43 co-pathology; however, because phospho-TDP-43 antibodies (such as pS409/410) are generally more sensitive for pathological TDP-43,^41^ particularly in LATE-NC, our approach may underestimate low-burden pathology. A limitation of the data-driven modelling of disease stage was the absence of early stage, incidental PSP cases, which are rare in the QSBB cohort. Strengths include the large number of patients, the availability of genetic and detailed longitudinal clinical data enabling standardized clinical phenotyping, and quantitative assessment of regional tau pathology using digital pathology methods that reduce inter- and intra-rater bias.

In summary, our results support a model in which PSP tau pathology distribution is influenced by spread along anatomical pathways and by intrinsic regional vulnerability, with the latter potentially contributing to early PSP tau pathology in distant brain regions. This study also provides additional longitudinal clinical data on a large cohort of PSP cases, presents novel anatomical reference standards for the expected regional distribution of tau pathology across MDS-defined PSP phenotypes, and characterizes the relationship between tau pathology and survival, with translational implications for studies evaluating therapies targeting tau removal and *in vivo* tau imaging in PSP.^3,74^ Digital pathology methods together with advances in machine learning algorithms hold the potential for performing large-scale, unbiased, and cell-specific quantification of primary tau pathology and co-pathologies across multiple clinicopathological cohorts. Integration of such data with region- and cell-specific gene expression readouts and inclusion as quantitative phenotypic traits in genome-wide association studies will further improve our understanding of the molecular drivers of tau propagation patterns and phenotypic diversity in PSP.

## Supplementary Material

awag131_Supplementary_Data

## Data Availability

Anonymized clinical and quantitative tau pathology data may be shared upon reasonable request by qualified investigators for purposes of replicating procedures and results, if in compliance with ethical approval. All custom Python and Groovy scripts used for the quantitative digital pathology pipeline are openly available in a public GitHub repository (https://github.com/JaunmuktaneLab).
