# Supplementary material for: Data-driven modelling of tau pathology reveals distinct progressive supranuclear palsy subtypes
Source: Brain. 2026 Apr 13;149(9):3080–94. doi: 10.1093/brain/awag131 (PMC13548875; doi:10.1093/brain/awag131)
Supplement: Supplementary file 1 [file awag131_supplementary_data.pdf]

Supplementary Table 1 Abbreviations and names of brain regions of interest

Region of interest		% missing	Region of interest		% missing
SFG-GM[a]	Superior frontal gyrus GM	0.4	CA1	Cornu ammonis subregion 1	0.8
SFG-WM[a]	Superior frontal gyrus WM	0.4	CA2	Cornu ammonis subregion 2	0.8
MFG-GM	Middle frontal gyrus GM	0.4	CA3	Cornu ammonis subregion 3	1.3
MFG-WM	Middle frontal gyrus WM	0.4	CA4	Cornu ammonis subregion 4	0.4
PCG-GM[a]	Precentral gyrus GM	1.3	DENT	Dentate gyrus	0.4
PCG-WM[a]	Precentral gyrus WM	1.3	GLOB_PALL[a]	Globus pallidus	0.4
SPL-GM[a]	Superior parietal lobule GM	0.4	CAU[a]	Caudate	0.8
SPL-WM[a]	Superior parietal lobule WM	0.4	INT-CAP[a]	Internal capsule	0.4
IPL-GM	Inferior parietal lobule GM	0.4	PUT	Putamen	0.4
IPL-WM	Inferior parietal lobule WM	0.4	DEEP-WM	Centrum semiovale WM	5.9
STG-GM	Superior temporal gyrus GM	0.4	LAT-CP	Lateral cerebral peduncle	6.8
STG-WM	Superior temporal gyrus WM	0.4	CENT-CP	Central cerebral peduncle	6.8
MTG-GM[a]	Middle temporal gyrus GM	0.4	MED-CP	Medial cerebral peduncle	8.5
MTG-WM[a]	Middle temporal gyrus WM	0.4	SUB_NIGRA[a]	Substantia nigra	4.2
FG-GM	Fusiform gyrus GM	0.8	TEGMENT[a]	Midbrain tegmentum	5.1
FG-WM	Fusiform gyrus WM	3.0	RED_NUC[a]	Red nucleus	5.9
ENTC	Entorhinal cortex	0.4	SUP_COLL	Superior colliculus	8.5
TRANSENTC	Transentorhinal cortex	0.4	PERIV-GM	Periventricular GM	10.2
PARASUB	Parasubiculum	1.3	CBM-DN	Cerebellar dentate nucleus	0.4
SUB	Subiculum	0.8	CBM-WM[a]	Cerebellar WM	0.4
PHG-WM	Parahippocampal gyrus WM	0.4			

[a]Regions of interest used for SuStaIn analyses. GM: grey matter; WM: white matter.

Supplementary Table 2 Brain region composites and constituent regions of interest

Brain region composites and constituent regions of interest	
Cortical GM	SFG-GM, MFG-GM, PCG-GM, SPL-GM, IPL-GM, STG-GM, MTG-GM
Cortical WM	SFG-WM, MFG-WM, PCG-WM, SPL-WM, IPL-WM, STG-WM, MTG-WM
Limbic GM	FG-GM, ENTC, TRANSENTC, PARASUB, SUB, CA1, CA2, CA3, CA4, DENT
Limbic WM	FG-WM, PHG-WM
Basal ganglia	GLOB_PALL, CAU, PUT
Deep WM	INT-CAP, DEEP-WM
Midbrain GM	TEGMENT, RED_NUC
Cerebral peduncle	LAT-CP, CENT-CP, MED-CP
Dentate nucleus	CBM-DN
Cerebellar WM	CBM-WM

Supplementary Table 3 Distribution of Thal, Braak and Braak, and CERAD scores across PSP clinical phenotypes

	**RS** (*n*=105)	**P** (*n*=49)	**CBS** (*n*=21)	**PI** (*n*=18)	**F** (*n*=13)	**SL** (*n*=8)	**PGF** (*n*=8)	***P*** **(adj.)**[a]
**Thal phase**								0.274
0	32 (30.5)	14 (28.6)	8 (38.1)	2 (11.1)	5 (41.7)	2 (25.0)	4 (50.0)	
1	20 (19.0)	7 (14.3)	2 (9.5)	5 (27.8)	2 (16.7)	2 (25.0)	2 (25.0)	
2	24 (22.9)	12 (24.5)	3 (14.3)	4 (22.2)	3 (25.0)	1 (12.5)	1 (12.5)	
3	23 (21.9)	7 (14.3)	7 (33.3)	5 (27.8)	2 (16.7)	0 (0.0)	0 (0.0)	
4	2 (1.9)	5 (10.2)	1 (4.8)	0 (0.0)	0 (0.0)	2 (25.0)	1 (12.5)	
5	4 (3.8)	4 (8.2)	0 (0.0)	2 (11.1)	0 (0.0)	1 (12.5)	0 (0.0)	
**Braak-NFT**								0.017
0	21 (20.0)	3 (6.1)	3 (14.3)	3 (16.7)	3 (25.0)	1 (12.5)	3 (37.5)	
1	21 (20.0)	8 (16.3)	3 (14.3)	4 (22.2)	1 (8.3)	1 (12.5)	3 (37.5)	
2	52 (49.5)	25 (51.0)	11 (52.4)	8 (44.4)	6 (50.0)	4 (50.0)	1 (12.5)	
3	10 (9.5)	4 (8.2)	4 (19.0)	3 (16.7)	0 (0.0)	0 (0.0)	0 (0.0)	
4	1 (1.0)	6 (12.2)	0 (0.0)	0 (0.0)	1 (8.3)	2 (25.0)	1 (12.5)	P vs RS
5	0 (0.0)	3 (6.1)	0 (0.0)	0 (0.0)	1 (8.3)	0 (0.0)	0 (0.0)	
**CERAD score**								0.058
0	67 (63.8)	24 (49.0)	13 (61.9)	7 (38.9)	8 (66.7)	4 (50.0)	6 (75.0)	
1	34 (32.4)	14 (28.6)	6 (28.6)	8 (44.4)	3 (25.0)	4 (50.0)	1 (12.5)	
2	4 (3.8)	11 (22.4)	2 (9.5)	3 (16.7)	1 (8.3)	0 (0.0)	1 (12.5)	

Data shown as number of patients (percentage).

[a]adjusted significant pairwise comparisons.

Braak-NFT stage 6 and CERAD score 3 cases were not observed in this cohort.

NFT: neurofibrillary tangle; CERAD: Consortium to Establish a Registry for Alzheimer's Disease neuritic plaque scores.

Supplementary Table 4 Univariate and adjusted Cox regression analysis for survival in PSP

Variable	N (%)	HR (95% CI)	*P*	*P* BH	Adj. HR (95% CI)	Adj. *P*	Adj. *P* BH
SLC2A13 (A)	82 (42)	0.99 (0.74 - 1.32)	0.932	0.949	1.06 (0.76 - 1.48)	0.724	0.789
TRIM11 (A)	33 (17)	1.08 (0.74 - 1.58)	0.705	0.800	1.08 (0.70 - 1.64)	0.733	0.789
MAPT H2	16 (8)	1.09 (0.65 - 1.83)	0.734	0.801	0.73 (0.40 - 1.33)	0.299	0.433
APOE ε4	41 (21)	1.11 (0.78 - 1.58)	0.578	0.674	1.14 (0.75 - 1.71) [HR@t]	0.540	0.648
Female sex	86 (36)	0.88 (0.67 - 1.14)	0.326	0.403	0.86 (0.64 - 1.15)	0.320	0.448
Onset age	236 (100)	1.03 (1.02 - 1.05)	<0.001	<0.001	1.04 (1.01 - 1.06) [HR@t]	<0.001	0.004
L-dopa response	57 (30)	0.86 (0.63 - 1.18)	0.365	0.438	0.95 (0.67 - 1.35)	0.767	0.805
Sustained L-dopa response	12 (6)	0.59 (0.33 - 1.06)	0.078	0.121	0.72 (0.42 - 1.26) [HR@t]	0.253	0.389
Visual symptoms	50 (21)	1.63 (1.19 - 2.25)	0.003	0.006	1.15 (0.79 - 1.66) [HR@t]	0.474	0.636
Blepharospasm/AEO	15 (6)	1.09 (0.62 - 1.90)	0.770	0.809	0.64 (0.33 - 1.23) [HR@t]	0.181	0.345
vSNGP	78 (33)	4.04 (2.98 - 5.47)	<0.001	<0.001	2.17 (1.46 - 3.24)	<0.001	0.001
Falls	185 (78)	2.00 (1.46 - 2.75)	<0.001	<0.001	1.34 (0.86 - 2.09)	0.203	0.370
Gait freezing	25 (11)	0.71 (0.46 - 1.08)	0.108	0.157	0.83 (0.47 - 1.46) [HR@t]	0.511	0.648
Axial rigidity	58 (25)	2.44 (1.80 - 3.31)	<0.001	<0.001	1.59 (1.13 - 2.24)	0.008	0.030
Limb rigidity	124 (53)	1.92 (1.48 - 2.50)	<0.001	<0.001	2.09 (1.50 - 2.91)	<0.001	<0.001
Bradykinesia	142 (60)	1.68 (1.29 - 2.19)	<0.001	<0.001	1.95 (1.39 - 2.75)	<0.001	<0.001
Tremor	62 (26)	0.95 (0.71 - 1.27)	0.744	0.801	1.22 (0.88 - 1.68)	0.235	0.379
Dysexecutive syndrome	69 (29)	1.93 (1.44 - 2.60)	<0.001	<0.001	1.45 (1.06 - 2.00)	0.022	0.061
Reduced verbal fluency	59 (25)	1.78 (1.31 - 2.41)	<0.001	<0.001	1.42 (1.01 - 2.00)	0.042	0.093
Apathy	42 (18)	1.19 (0.85 - 1.67)	0.313	0.398	1.18 (0.74 - 1.88) [HR@t]	0.484	0.636
Bradyphrenia	51 (22)	2.06 (1.49 - 2.85)	<0.001	<0.001	1.44 (1.01 - 2.06)	0.042	0.093
Behavioural change	71 (30)	1.58 (1.19 - 2.10)	0.001	0.003	1.27 (0.92 - 1.76)	0.148	0.311
Apraxia	27 (11)	1.71 (1.13 - 2.57)	0.011	0.021	2.00 (1.14 - 3.52)	0.016	0.056
Alien limb	12 (5)	1.60 (0.89 - 2.88)	0.115	0.160	1.60 (0.71 - 3.63)	0.259	0.389
Cortical sensory signs	9 (4)	1.02 (0.52 - 1.99)	0.949	0.949	0.54 (0.20 - 1.44)	0.215	0.376
Myoclonus	5 (2)	2.19 (0.90 - 5.34)	0.086	0.128	1.82 (0.69 - 4.76)	0.224	0.376
Frontalis overactivity	24 (10)	1.79 (1.17 - 2.74)	0.007	0.015	1.01 (0.60 - 1.69) [HR@t]	0.973	0.973
Cervical/limb dystonia	26 (11)	1.50 (0.99 - 2.26)	0.053	0.086	1.15 (0.74 - 1.80)	0.540	0.648
Pyramidal signs	24 (10)	1.40 (0.92 - 2.15)	0.118	0.160	1.14 (0.72 - 1.80)	0.583	0.680
Cerebellar signs	8 (3)	1.61 (0.79 - 3.28)	0.190	0.250	1.20 (0.55 - 2.61) [HR@t]	0.647	0.734
Constipation	39 (17)	2.34 (1.65 - 3.33)	<0.001	<0.001	1.94 (1.28 - 2.94) [HR@t]	0.002	0.008
Urinary symptoms	87 (37)	2.34 (1.76 - 3.11)	<0.001	<0.001	1.82 (1.33 - 2.48)	<0.001	0.001
Orthostatic hypotension	9 (4)	2.08 (1.06 - 4.08)	0.032	0.054	0.94 (0.43 - 2.03)	0.870	0.891
Depression	48 (20)	1.52 (1.10 - 2.09)	0.011	0.021	1.47 (1.05 - 2.07)	0.027	0.066
Visual hallucinations	13 (6)	2.72 (1.54 - 4.81)	<0.001	0.001	2.01 (1.12 - 3.59)	0.019	0.056
Clinical RBD	7 (3)	2.61 (1.22 - 5.57)	0.013	0.023	2.42 (1.11 - 5.29)	0.026	0.066
Insomnia	33 (14)	1.99 (1.37 - 2.89)	<0.001	<0.001	2.02 (1.36 - 2.99)	<0.001	0.003
Dementia	18 (8)	1.86 (1.15 - 3.02)	0.012	0.022	1.46 (0.86 - 2.49)	0.162	0.324
Urinary catheter	7 (3)	5.05 (2.33 - 10.94)	<0.001	<0.001	3.17 (1.22 - 8.24)	0.018	0.056
Wheelchair	22 (9)	4.19 (2.65 - 6.63)	<0.001	<0.001	3.62 (2.18 - 6.01)	<0.001	<0.001
Care requirement	41 (17)	6.44 (4.36 - 9.50)	<0.001	<0.001	3.70 (2.40 - 5.69)	<0.001	<0.001
Severe bulbar symptoms	12 (5)	10.54 (5.69 - 19.54)	<0.001	<0.001	5.21 (2.75 - 9.86)	<0.001	<0.001

The table summarises Cox proportional hazards regression analyses of survival. For each predictor, we report the number (N) and percentage of patients with the feature present within the first 3 years from symptoms onset, crude and adjusted hazard ratios (HR), 95%

confidence intervals (CI), raw *P*-values (*P*), and Benjamini–Hochberg corrected *P*-values (*P* BH). Crude estimates are from univariate Cox models. Adjusted estimates are from separate multivariable Cox models including the predictor of interest plus sex and onset age, with stratification by phenotype. Proportional hazards assumptions were assessed using Schoenfeld residuals; violations were handled by using time-varying coefficients (denoted by [HR@t]), with HRs for time-varying terms evaluated at the median disease duration.
Visual symptoms: blurred vision, diplopia or photophobia; AEO: apraxia of eyelid opening; vSNGP: vertical supranuclear gaze pa lsy; RBD: REM sleep behaviour disorder; severe bulbar symptoms: severe dysarthria or dysphagia; GM: grey matter; WM: white matter.

Supplementary Table 5 Region-wise associations between tau pathology and survival

Region	N	β	95% CI	*P*	BH *P*	Region	N	β	95% CI	*P*	BH *P*
SFG-GM	221	-0.012	(-0.025, 0.002)	0.093	0.190	ENTC	221	0.003	(-0.001, 0.007)	0.110	0.206
SFG-WM	221	-0.011	(-0.031, 0.010)	0.310	0.374	TRANSENTC	221	0.003	(-0.001, 0.007)	0.188	0.316
MFG-GM	221	-0.017	(-0.032, -0.001)	0.035	0.096	FG-GM	220	0.007	(0.001, 0.013)	0.034	0.096
MFG-WM	221	-0.018	(-0.045, 0.010)	0.215	0.316	FG-WM	217	0.017	(-0.011, 0.047)	0.239	0.316
PCG-GM	220	-0.004	(-0.012, 0.004)	0.287	0.356	PHG-WM	221	0.006	(-0.006, 0.018)	0.344	0.392
PCG-WM	220	-0.023	(-0.040, -0.005)	0.008	0.031	GLOB_PALL	221	-0.021	(-0.031, -0.011)	0.000	0.001
STG-GM	221	0.005	(-0.007, 0.017)	0.412	0.441	CAU	220	-0.021	(-0.034, -0.007)	0.002	0.015
STG-WM	221	0.021	(-0.011, 0.055)	0.214	0.316	INT-CAP	221	-0.011	(-0.022, -0.001)	0.035	0.096
MTG-GM	221	0.011	(0.001, 0.022)	0.043	0.104	PUT	221	-0.018	(-0.028, -0.007)	0.002	0.013
MTG-WM	221	0.038	(-0.016, 0.093)	0.178	0.316	DEEP-WM	208	-0.020	(-0.033, -0.005)	0.007	0.031
SPL-GM	221	-0.007	(-0.019, 0.005)	0.224	0.316	LAT-CP	206	-0.014	(-0.038, 0.012)	0.279	0.356
SPL-WM	221	-0.029	(-0.052, -0.006)	0.012	0.040	CENT-CP	206	-0.009	(-0.031, 0.014)	0.420	0.441
IPL-GM	221	0.001	(-0.015, 0.017)	0.950	0.950	MED-CP	202	0.009	(-0.005, 0.023)	0.235	0.316
IPL-WM	221	-0.036	(-0.079, 0.008)	0.108	0.206	SUB_NIGRA	212	-0.019	(-0.032, -0.006)	0.005	0.026
CA1	220	0.003	(-0.001, 0.007)	0.206	0.316	TEGMENT	210	-0.014	(-0.023, -0.006)	0.001	0.012
CA2	220	0.005	(-0.001, 0.010)	0.087	0.188	RED_NUC	208	-0.014	(-0.022, -0.007)	0.000	0.003
CA3	220	0.009	(0.001, 0.018)	0.039	0.101	SUP_COLL	202	-0.010	(-0.017, -0.003)	0.008	0.031
CA4	221	0.009	(-0.009, 0.028)	0.343	0.392	PERIV-GM	200	-0.017	(-0.028, -0.007)	0.001	0.012
DENT	221	0.004	(-0.005, 0.015)	0.358	0.396	CBM-DN	221	-0.062	(-0.090, -0.033)	0.000	0.001
SUB	220	0.002	(-0.001, 0.006)	0.237	0.316	CBM-WM	221	-0.008	(-0.031, 0.016)	0.497	0.509
PARASUB	219	0.007	(0.000, 0.014)	0.056	0.128						

This table presents the results of gamma generalized linear models (GLMs) with log link assessing the association between regional tau pathology and survival. Models were conducted separately for each region. All models were adjusted for age at onset, sex, clinical phenotype, and NIA-ABC score. For each region, beta coefficient (β), 95% confidence interval (CI), raw *P*-value (*P*), and Benjamini–Hochberg adjusted *P*-value (BH *P*) are shown.

Supplementary Table 6 Regional tau pathology burden by phenotype

Region	PSP-RS		PSP-P		PSP-CBS		PSP-PI		PSP-F		PSP-SL		PSP-PGF	
	n	Mean (sd)	*n*	Mean (sd)	*n*	Mean (sd)	*n*	Mean (sd)	*n*	Mean (sd)	*n*	Mean (sd)	*n*	Mean (sd)
SFG-GM	105	2.1 (2.1)	49	2.9 (5.2)	21	3.9 (4.3)	18	1.4 (1.5)	12	2.9 (2.1)	8	8.8 (7.4)	8	0.8 (0.9)
SFG-WM	105	1.5 (1.9)	49	0.9 (1.2)	21	2.3 (2.4)	18	0.7 (0.7)	12	2.7 (4.8)	8	4.3 (2.6)	8	0.4 (0.6)
MFG-GM	105	1.5 (1.8)	49	2.2 (4.7)	21	2.6 (3.2)	18	0.8 (0.8)	12	2.1 (2.0)	8	5.4 (5.8)	8	0.4 (0.4)
MFG-WM	105	1.1 (1.5)	49	0.6 (1.0)	21	2.1 (2.2)	18	0.5 (0.5)	12	1.9 (2.7)	8	2.4 (1.0)	8	0.3 (0.3)
PCG-GM	104	4.8 (5.5)	49	3.1 (5.8)	21	9.6 (7.6)	18	3.1 (4.1)	12	6.0 (6.5)	8	8.6 (5.8)	8	1.7 (3.3)
PCG-WM	104	1.5 (2.2)	49	0.8 (1.4)	21	6.1 (4.9)	18	1.5 (3.2)	12	1.5 (1.6)	8	3.1 (3.5)	8	0.2 (0.4)
STG-GM	105	0.7 (1.4)	49	3.0 (8.3)	21	1.1 (1.5)	18	0.4 (0.2)	12	4.0 (9.3)	8	12.7 (15.5)	8	0.8 (1.7)
STG-WM	105	0.3 (0.5)	49	0.6 (1.5)	21	0.7 (1.1)	18	0.2 (0.2)	12	1.2 (2.4)	8	5.4 (5.4)	8	0.1 (0.2)
MTG-GM	105	0.8 (2.9)	49	4.1 (9.9)	21	0.6 (0.6)	18	0.8 (1.5)	12	3.6 (9.4)	8	10.8 (16.0)	8	2.2 (5.7)
MTG-WM	105	0.3 (0.4)	49	0.6 (0.9)	21	0.3 (0.4)	18	0.5 (1.3)	12	0.9 (1.5)	8	3.1 (2.9)	8	0.2 (0.2)
SPL-GM	105	2.1 (3.8)	49	1.8 (3.5)	21	3.7 (3.1)	18	1.4 (2.1)	12	2.0 (2.6)	8	5.1 (7.4)	8	1.6 (3.3)
SPL-WM	105	1.2 (1.6)	49	0.9 (1.7)	21	3.2 (3.1)	18	0.8 (1.2)	12	1.5 (2.7)	8	2.3 (1.8)	8	0.8 (1.8)
IPL-GM	105	1.3 (1.9)	49	2.2 (5.1)	21	1.9 (2.0)	18	0.6 (0.7)	12	2.9 (6.8)	8	4.8 (7.9)	8	0.5 (0.8)
IPL-WM	105	0.8 (1.0)	49	0.4 (0.5)	21	1.3 (1.5)	18	0.4 (0.6)	12	1.2 (1.8)	8	1.8 (0.8)	8	0.3 (0.5)
CA1	104	6.7 (10.1)	49	14.4 (14.3)	21	7.1 (8.3)	18	9.4 (11.4)	12	8.0 (11.8)	8	18.6 (16.1)	8	8.0 (12.9)
CA2	104	4.0 (6.8)	49	10.1 (11.6)	21	4.0 (7.0)	18	4.4 (6.2)	12	7.5 (11.5)	8	21.1 (17.0)	8	7.2 (9.7)
CA3	104	2.2 (4.6)	49	4.0 (5.3)	21	2.3 (5.0)	18	2.1 (4.6)	12	4.0 (6.1)	8	9.3 (7.7)	8	3.4 (5.2)
CA4	105	1.2 (2.2)	49	2.0 (2.6)	21	1.2 (3.3)	18	1.0 (1.5)	12	1.5 (1.9)	8	3.8 (3.6)	8	1.0 (1.4)
DENT	105	1.7 (3.0)	49	4.4 (7.5)	21	2.5 (6.7)	18	2.8 (4.3)	12	2.2 (3.9)	8	7.5 (8.8)	8	1.7 (2.4)
SUB	104	7.1 (10.9)	49	17.3 (16.1)	21	8.3 (9.9)	18	14.5 (14.2)	12	9.7 (13.5)	8	18.2 (16.1)	8	9.7 (17.4)
PARASUB	104	2.6 (4.9)	48	7.0 (10.3)	21	3.1 (5.9)	18	3.2 (4.4)	12	3.6 (5.0)	8	12.5 (14.6)	8	4.4 (8.7)
ENTC	105	9.4 (10.8)	49	15.8 (14.4)	21	11.4 (13.2)	18	12.2 (10.1)	12	8.9 (10.9)	8	21.8 (18.8)	8	10.1 (12.2)
TRANSENTC	105	7.1 (10.5)	49	16.6 (18.2)	21	9.9 (12.9)	18	11.8 (11.3)	12	11.9 (15.0)	8	17.5 (19.1)	8	9.6 (16.1)
FG-GM	104	1.9 (5.2)	49	9.8 (15.6)	21	1.6 (1.9)	18	2.9 (5.4)	12	6.8 (13.0)	8	12.1 (19.3)	8	4.9 (12.8)
FG-WM	102	0.4 (0.8)	49	1.3 (2.0)	21	0.4 (0.4)	17	1.0 (2.9)	12	1.7 (3.8)	8	2.3 (2.9)	8	0.7 (1.5)
PHG-WM	105	1.7 (2.8)	49	4.1 (5.1)	21	1.7 (2.4)	18	3.0 (6.3)	12	2.5 (3.0)	8	4.9 (5.3)	8	2.1 (3.3)
GLOB_PALL	105	4.2 (4.0)	49	3.6 (6.8)	21	3.1 (3.8)	18	2.6 (2.3)	12	4.0 (4.8)	8	3.5 (2.1)	8	2.9 (1.6)
CAU	104	3.3 (2.5)	49	3.1 (5.2)	21	3.3 (3.4)	18	2.3 (2.2)	12	4.0 (3.2)	8	5.1 (2.1)	8	1.9 (2.0)
INT-CAP	105	4.8 (4.4)	49	2.9 (4.1)	21	2.9 (2.5)	18	3.2 (2.8)	12	5.5 (6.1)	8	6.7 (4.1)	8	1.7 (1.8)
PUT	105	2.5 (2.5)	49	2.7 (6.2)	21	4.4 (5.6)	18	1.6 (1.5)	12	2.9 (3.6)	8	2.8 (1.4)	8	1.3 (1.6)
DEEP-WM	98	2.0 (2.5)	49	2.0 (5.0)	19	2.3 (2.3)	17	1.1 (1.0)	11	3.1 (3.4)	7	4.0 (1.8)	7	1.2 (2.3)
LAT-CP	98	1.4 (1.7)	46	0.9 (1.8)	20	1.9 (1.8)	18	1.6 (2.8)	11	1.1 (0.9)	8	2.1 (1.3)	5	0.4 (0.4)
CENT-CP	100	2.1 (2.4)	46	0.9 (1.2)	19	3.5 (2.4)	17	1.9 (2.1)	11	1.5 (1.0)	8	3.0 (1.2)	5	0.7 (0.6)
MED-CP	98	3.4 (3.7)	45	1.4 (1.8)	18	3.0 (3.3)	17	2.8 (3.8)	11	3.4 (3.3)	8	5.6 (3.6)	5	0.9 (0.8)

SUB_NIGRA	101	5.2 (4.1)	48	2.6 (2.0)	20	4.1 (2.7)	18	3.3 (3.0)	11	4.7 (3.2)	8	5.4 (3.0)	6	3.1 (0.9)
TEGMENT	101	9.1 (6.2)	46	5.2 (3.4)	20	6.6 (3.3)	18	6.2 (4.4)	11	9.2 (7.5)	8	7.4 (3.6)	6	5.7 (3.1)
RED_NUC	100	10.0 (6.8)	46	5.0 (4.1)	20	9.4 (6.2)	18	6.9 (5.7)	11	8.9 (5.2)	8	9.6 (4.5)	5	4.3 (5.0)
SUP_COLL	98	10.2 (7.4)	43	6.3 (4.2)	19	8.1 (5.5)	18	5.9 (4.7)	11	10.1 (6.3)	7	11.3 (5.6)	6	7.6 (3.0)
PERIV-GM	97	7.2 (5.0)	42	4.9 (3.0)	19	4.8 (2.9)	18	4.9 (4.2)	11	5.9 (3.8)	7	7.7 (3.1)	6	6.6 (3.9)
CBM-DN	105	2.0 (1.6)	49	1.2 (0.9)	21	2.0 (1.3)	18	1.5 (1.0)	12	2.1 (1.1)	8	1.4 (1.0)	8	1.0 (1.9)
CBM-WM	105	2.0 (2.3)	49	0.6 (0.6)	21	1.5 (2.2)	18	1.3 (1.6)	12	2.1 (1.9)	8	0.9 (0.9)	8	0.4 (0.8)

Mean tau pathology burden is shown for each region of interest grouped by clinical phenotype.
n: number of cases. sd: standard deviation.

Supplementary Table 7 Association between regional tau pathology composites and ages at onset and death

Tau pathology regional	Age at death ρ	*P*	Age at onset ρ	*P*
Cortical-GM	-0.051	.468	0.026	.710
Limbic-GM	0.235	<.001	0.257	<.001
Basal ganglia-GM	-0.088	.205	0.020	.769
Midbrain-GM	-0.273	<.001	-0.186	.007
Dentate nucleus-GM	-0.031	.654	0.097	.164
Cortical-WM	-.04	.568	0.045	.521
Limbic-WM	0.217	.002	0.232	<.001
Deep-WM	-0.248	<.001	-0.176	.011
Cerebral peduncle-WM	-0.056	.419	-0.051	.458
Cerebellum-WM	-0.143	.039	-0.102	.141

Spearman correlations between age and regional tau pathology composites. All variables were adjusted for sex, NIA-ABC score, and clinical phenotype using linear models. Spearman correlation coefficients were computed between the residuals.

Supplementary Table 8 Clinical, genetic, and neuropathological characteristics across SuStaIn subtypes

	Subtype 1	Subtype 2	Subtype 3	*P*	adj.[a]
**Female sex**	53 (38.4)	9 (30.0)	8 (34.8)	0.674	
**AAO**	66.86 (7.01)	68.97 (7.00)	68.38 (6.25)	0.244	
**Survival**	7.56 (2.77)	8.88 (3.45)	6.84 (2.34)	0.024	ST2 vs ST3
**AAD**	74.41 (6.66)	77.85 (7.22)	75.23 (6.96)	0.044	ST1 vs ST2
**NIA-ABC score**				0.403	
0	50 (36.2)	9 (30.0)	11 (47.8)		
1	88 (63.8)	21 (70.0)	12 (52.2)		
**Thal phase**				0.310	
0	49 (35.5)	9 (30.0)	10 (43.5)		
1	26 (18.8)	7 (23.3)	5 (21.7)		
2	35 (25.4)	6 (20.0)	3 (13.0)		
3	25 (18.1)	4 (13.3)	4 (17.4)		
4	1 (0.7)	2 (6.7)			
5	2 (1.4)	2 (6.7)	1 (4.3)		
**CERAD neuritic plaque score**				0.557	
0	94 (68.1)	16 (53.3)	16 (69.6)		
1	40 (29.0)	12 (40.0)	6 (26.1)		
2	4 (2.9)	2 (6.7)	1 (4.3)		
**Braak-NFT**				0.008	
0	27 (19.6)	2 (6.7)	6 (26.1)		
1	30 (21.7)	4 (13.3)	5 (21.7)		
2	76 (55.1)	17 (56.7)	10 (43.5)		
3	5 (3.6)	5 (16.7)	2 (8.7)		
4	0 (0)	2 (6.7)	0 (0)		
**Braak-LP**				0. 902	
0	120 (87.0)	27 (90.0)	22 (95.7)		
1	1 (0.7)	0 (0)	1 (4.3)		
2	3 (2.2)	0 (0)	0 (0)		
3	3 (2.2)	1 (3.3)	0 (0)		
4	6 (4.3)	1 (3.3)	0 (0)		
5	3 (2.2)	1 (3.3)	0 (0)		
6	2 (1.4)	0 (0)	0 (0)		
**A-P LP**				0. 360	
No	134 (97.1)	28 (93.3)	22 (95.7)		
Yes	4 (2.9)	2 (6.7)	1 (4.3)		
**TDP-43**				0.005	
None	133 (96.4)	22 (73.3)	21 (91.3)		
1	0 (0)	1 (3.3)	0 (0)		
≥ 2	3 (2.2)	5 (16.7)	1 (4.3)		
NOS	2 (1.4)	2 (6.7)	1 (4.3)		
***APOE* ε4**				0.481	
No	89 (80.9)	22 (84.6)	17 (94.4)		
Yes	21 (19.1)	4 (15.4)	1 (5.6)		
***MAPT* H2**				0.077	
No	105 (95.5)	22 (84.6)	16 (88.9)		
Yes	5 (4.5)	4 (15.4)	2 (11.1)		
***TRIM11* (A)**				0.794	
No	89 (80.9)	22 (84.6)	16 (88.9)		
Yes	21 (19.1)	4 (15.4)	2 (11.1)		
***SLC2A13* (A)**					
No	66 (60.0)	16 (61.5)	11 (61.1)	0.987	
Yes	44 (40.0)	10 (38.5)	7 (38.9)		
**Clinical phenotype**				<0.001	
PSP-RS	79 (57.2)	7 (23.3)	6 (26.1)		ST1 vs ST2:
PSP-P	21 (15.2)	9 (30.0)	1 (4.3)		RS, SL
PSP-PI	10 (7.2)	2 (6.7)	2 (8.7)		
PSP-PGF	5 (3.6)	0 (0)	1 (4.3)		ST1 vs ST3:
PSP-CBS	5 (3.6)	2 (6.7)	12 (52.2)		RS, CBS
PSP-F	6 (4.3)	3 (10.0)	1 (4.3)		
PSP-SL	1 (0.7)	5 (16.7)	0 (0)		ST2 vs ST3:
No MDS phenotype	11 (8.0)	2 (6.7)	0 (0)		CBS

Data shown as number of patients (percentage) for categorical variables and mean number of years (standard deviation) for continuous variables.

[a]adjusted significant pairwise comparisons.

ST: subtype; AAO: age at onset; AAD: age at death; NIA-ABC: National Institute on Aging–Alzheimer's Association ABC score; CERAD: Consortium to Establish a Registry for Alzheimer's Disease; NFT: neurofibrillary tangle; A-P: amygdala predominant; LP: Lewy pathology; LATE-NC: Limbic-predominant Age-related TDP-43 Encephalopathy-Neuropathological Change; NOS: not otherwise specified.

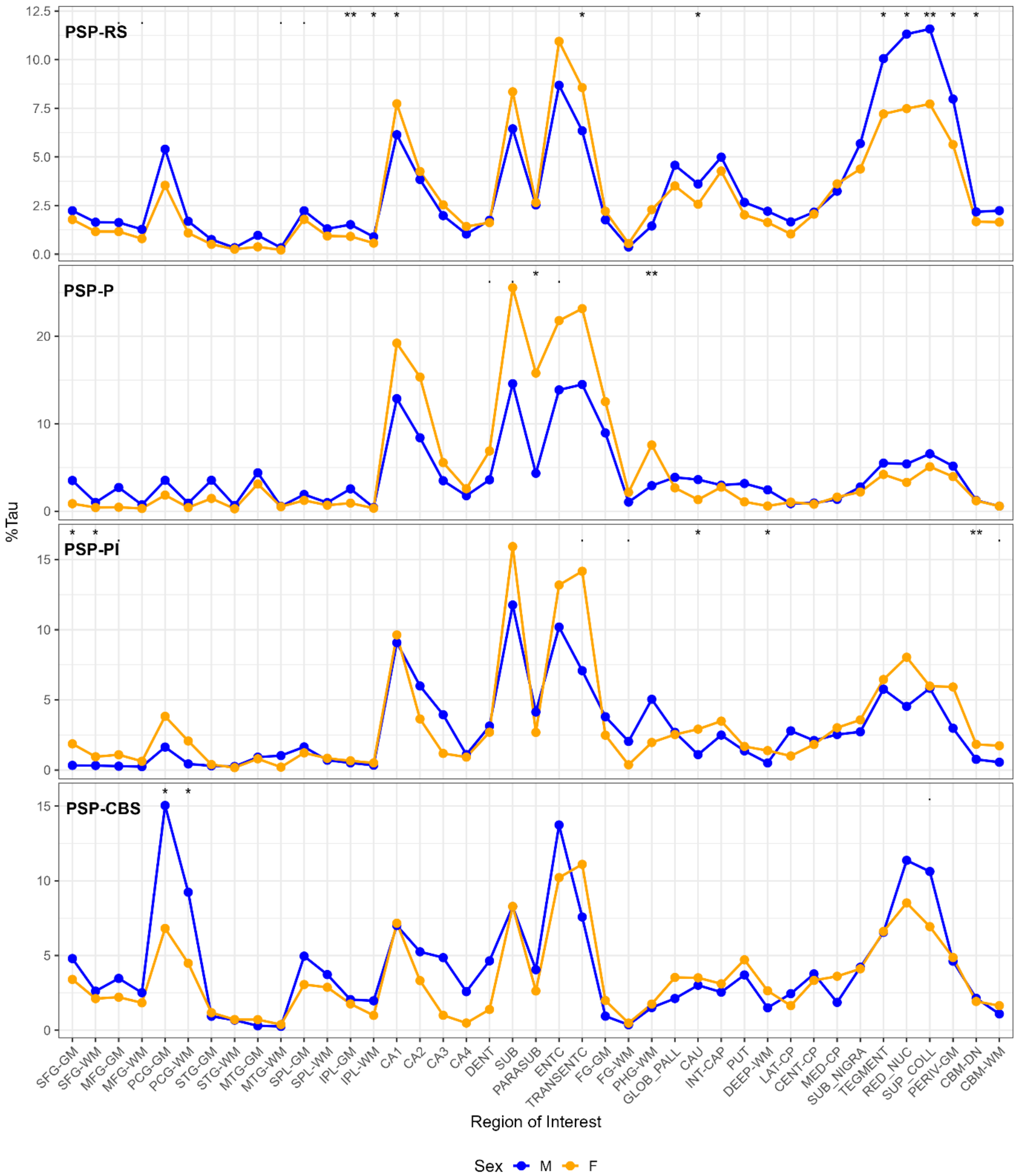

Supplementary Figure 1 Sex differences in regional tau pathology burden by phenotype. Line plots show mean tau pathology (%Tau) by region for male (blue) and female (orange) patients. Each panel represents one clinical phenotype. Asterisks indicate unadjusted pairwise Wilcoxon rank-sum test significance (*$P < 0.05$, **$P < 0.01$, ***$P < 0.001$). No comparisons survived Benjamini–Hochberg correction for multiple comparisons.

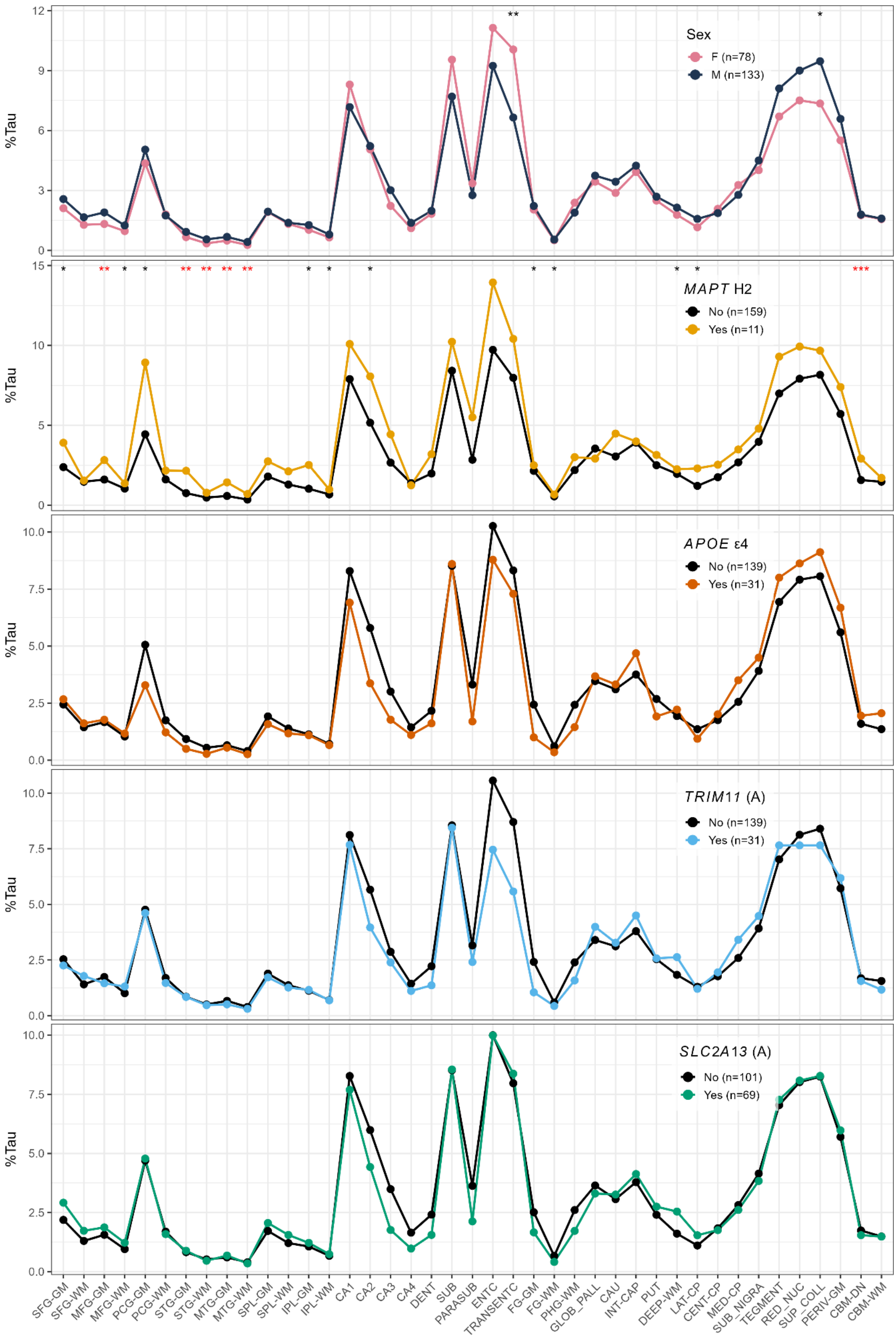

Supplementary Figure 2 Regional tau pathology by sex and genotype excluding patients with NIA–ABC score ≥ 2. The line plots show mean tau pathology burden (% Tau) across 41

brain regions for each category of sex, *MAPT* H2, *APOE* ε4, *TRIM11* (A), and *SLC2A13* (A). Error bars have been omitted for clarity. Asterisks indicate nominally significant differences between groups in individual regions (*$P < 0.05$, **$P < 0.01$, ***$P < 0.001$). Red significance symbols denote regions that remain significant following Benjamini–Hochberg correction for multiple comparisons. Sample sizes (*n*) for each group are indicated in the legends.

Clinical features and disease progression milestone[1,2,3,4]

Alien limb: The date of onset/first documentation of symptoms/signs of alien limb phenomenon.

Apathy: The date of onset/first documentation of reduced level of interest, initiative, and spontaneous activity; clearly apparent to informant or patient.

Apraxia: The date of onset/first documentation of orobuccal or limb apraxia.

Axial rigidity: The date of onset/first documentation of symptoms of axial rigidity or axial rigidity on examination.

Behavioural change: The date of onset/first documentation of impulsivity, disinhibition, or perseveration, e.g. socially inappropriate behaviours, overstuffing the mouth when eating, motor recklessness, applause sign, palilalia, echolalia.

Bradykinesia/hypokinesia: The date of onset/first documentation of symptoms of bradykinesia or bradykinesia/hypokinesia on examination.

Bradyphrenia: The date of onset/first documentation of slowed thinking; clearly apparent to informant or patient.

Blepharospasm or Apraxia of Eyelid Opening: The date of onset/first documentation of blepharospasm/AEO during either on physical examination or based on symptoms described by the patient or relative/carer.

Cerebellar signs: The date of first documentation of cerebellar signs on examination.

Cortical sensory signs: The date of onset/first documentation of cortical sensory deficits e.g. agraphesthesia, astereognosis.

Dementia: The date of onset/first documentation of cognitive impairment which affects the patient's ability to perform tasks of daily living.

Diplopia/blurred vision: The date of onset/first documentation of blurred vision or diplopia not explained by another cause.

Dysexecutive syndrome: The date of onset/first documentation of symptoms or signs of executive dysfunction including reverse digit span, Trails B or Stroop test, Luria sequence.

Dystonia: The date of onset/first documentation of symptoms of dystonia or dystonia observed on examination, including craniofacial, cervical or Limb dystonia.

Falls: The date when the patient has reached the milestone of ≥2 falls/year or documentation of "frequent" or "regular" falls.

Frontalis overactivity: The date of first documentation of frontalis overactivity by clinician.

Gait freezing: The date of onset/first documentation of symptoms of gait freezing (upon gait initiation, turning etc…) or gait freezing observed on examination.
Limb rigidity: The date of onset/first documentation of symptoms or limb rigidity on examination.
Myoclonus: The date of onset/first documentation of myoclonus or myoclonus observed on examination.
Orthostatic hypotension: date of onset of symptoms of orthostatic hypotension or asymptomatic postural drop in blood pressure ≥20/10mm/Hg.
Pyramidal signs: The date of first documentation of pyramidal signs on examination.
Reduced verbal fluency: The date of onset/first documentation of problems with verbal fluency either reported by the patient/carer or documented on examination.
REM sleep behaviour disorder: The date of onset/first documentation of body movement, emotional expression, or audible verbalisation of dream content during sleep time.
Severe dysarthria: The date when the patient's speech becomes unintelligible or requirement of communication aids.
Severe dysphagia (PEG): The date the patient was recommended to have a PEG/had PEG performed if former not available. In the event PEG tube placement was declined by patient, record the date it was recommended.
Tremor: The date of onset/first documentation of any rest, action, postural tremor either reported by the patient/carer or observed on examination.
Urinary catheter: The date the patient began to use a urinary catheter regularly.
Urinary dysfunction: The date of onset of lower urinary tract symptoms, including urinary urgency, frequency, nocturia, or incontinence.
Vertical supranuclear gaze palsy (vSNGP): The date of documentation of the first evidence of vertical supranuclear gaze palsy (O1).
Visual Hallucination: The date of onset/first documentation of visual hallucination.

References:

1. Grimm M-J, Respondek G, Stamelou M, et al. How to Apply the Movement Disorder Society Criteria for Diagnosis of Progressive Supranuclear Palsy. *Mov Disord* 2019; 34: 1228–32.

2. Höglinger GU, Respondek G, Stamelou M, et al. Clinical diagnosis of progressive supranuclear palsy: The movement disorder society criteria. *Mov Disord* 2017; 32: 853–64.

3. Jecmenica Lukic M, Kurz C, Respondek G, et al. Copathology in Progressive Supranuclear Palsy: Does It Matter? *Mov Disord* 2020; 35: 984–93.
4. O'Sullivan SS, Massey LA, Williams DR, et al. Clinical outcomes of progressive supranuclear palsy and multiple system atrophy. *Brain* 2008; 131: 1362–72.
